# Comparative Outcomes of Endoscopic, Minimally Invasive Surgical, and Open Necrosectomy in Necrotizing Pancreatitis: Evidence From a Network Meta‐Analysis

**DOI:** 10.1002/wjs.70406

**Published:** 2026-06-02

**Authors:** Ahmed Abdelsamad, Jawad Alqedra, Ibrahim Khalil, Mohammed Khaled Mohammed, Osama Elsayed Mohammed Selim, Amr Elserafy, Omar A. Ahmed, Mennatullah Mohsen, Ahmed Elsherif, Florian Gebauer, Khaled Ashraf Mohamed

**Affiliations:** ^1^ Department of Surgery II University of Witten/Herdecke Witten Germany; ^2^ Section Head of Robotic and Oncological Surgery Knappschaft Vest‐Hospital Recklinghausen Germany; ^3^ Faculty of Medicine Cairo University Cairo Egypt; ^4^ Faculty of Medicine Alexandria University Alexandria Egypt; ^5^ Department of Trauma and Emergency General Surgery University Hospitals Birmingham NHS Foundation Trust Birmingham UK; ^6^ Faculty of Medicine Ain Shams University Cairo Egypt; ^7^ Faculty of Medicine Al‐Azhar University Cairo for Girls Egypt; ^8^ St. Josef Hospital Bochum Bochum Germany; ^9^ Department of Gastrointestinal Surgery University of Helios Wuppertal Wuppertal Germany

**Keywords:** endoscopic trans‐gastric necrosectomy, minimally invasive surgical necrosectomy, necrotizing pancreatitis, network meta‐analysis, open necrosectomy

## Abstract

**Background:**

The optimal interventional approach for necrotizing pancreatitis remains debated. We performed a systematic review with network meta‐analysis and proportional meta‐analysis to compare endoscopic trans‐gastric necrosectomy (EN), minimally invasive surgical necrosectomy (MIN), and open necrosectomy (ON).

**Methods:**

Databases were searched from inception through March 2026. Eligible studies included randomized and observational comparative studies enrolling adults with necrotizing pancreatitis requiring necrosectomy and comparing ON, MIN, and/or EN. Nine binary outcomes were assessed in a frequentist random‐effects network meta‐analysis. Separate proportional meta‐analysis of single‐arm studies pooled mortality, clinical success, complications, and need for additional surgery. Risk of bias was assessed using RoB 2 and ROBINS‐I, and confidence in network estimates was assessed using CINeMA.

**Results:**

Thirty‐three studies were included. Nine comparative studies were included in the network meta‐analysis, and 24 single‐arm studies were analyzed separately. EN was associated with lower mortality, fewer complications, less new‐onset multiple organ failure, less exocrine insufficiency, fewer new ICU admissions, and better overall treatment ranking than ON. EN also outperformed MIN for complications and new‐onset multiple organ failure, whereas MIN was superior to ON for multiple organ failure, exocrine insufficiency, incisional hernia, and ICU admission. Bleeding, re‐intervention, and new‐onset diabetes did not differ significantly across techniques. In single‐arm pooling, EN and MIN showed comparable mortality, clinical success, complications, and need for additional surgery.

**Conclusions:**

Endoscopic necrosectomy appears to offer the most favorable overall profile for necrotizing pancreatitis, whereas minimally invasive surgical approaches also improve several outcomes compared with open necrosectomy. Open surgery should likely remain reserved for selected rescue situations.

## Graphical abstract

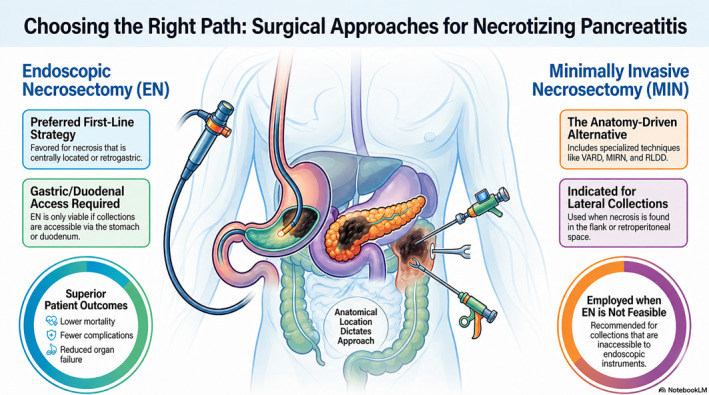

**Abbreviations**
CINeMA: confidence in network meta‐analysis
EN: endoscopic transgastric necrosectomy
GI: gastrointestinal
ICU: intensive care unit
iWON: infected walled‐off pancreatic necrosis
MD: mean difference
MIN: minimally invasive necrosectomy (VARD + Non‐VARD)
MIRN: minimal incision retroperitoneal necrosectomy (non‐VARD)
NMA: network meta‐analysis
Non‐MIN: (endoscopic approaches + open approach) (EN + ON)
Non‐VARD: (MIRN + RLDD + percutaneous drainage)
ON: open necrosectomy
RCTs: randomized clinical trials
RLDD: retroperitoneal laparoscopic debridement and drainage
SD: standard deviation
VARD: video‐assisted retroperitoneal debridement

## 1 Introduction

Necrotizing pancreatitis is a severe and potentially fatal disease, with mortality ranging from 10% to 50% depending on severity, organ failure, and infected necrosis [1]. Despite advances in intensive care, endoscopy, and surgery, it remains one of the most challenging entities in gastroenterology and surgical practice [2]. The condition is characterized by extensive pancreatic and peripancreatic tissue destruction, systemic inflammation, and a substantial risk of multi‐organ dysfunction [3]. A critical determinant of outcome is the timing and type of intervention [4]. Early necrosectomy before maturation of necrosis carries high morbidity and mortality [5]. Current consensus recommends delaying intervention by approximately three to 4 weeks until collections evolve into walled‐off necrosis, unless clinical deterioration mandates earlier treatment, such as in progressive sepsis, persistent organ failure, or gas‐containing collections [6]. Therefore, the interventional strategy should be tailored to the patient's clinical condition, anatomical distribution of necrosis, and necrotic burden, all of which influence feasibility and safety [7, 8].

Over recent decades, the management of infected walled‐off necrosis has shifted from routine open necrosectomy to a multidisciplinary step‐up strategy, escalating from the least to the most invasive modalities [9]. Percutaneous catheter drainage is often the initial step, achieving infection control and occasionally obviating the need for necrosectomy, although persistent solid debris frequently necessitates further intervention [10]. Endoscopic transluminal drainage, usually EUS‐guided, permits internal drainage through the stomach or duodenum and can be combined with direct endoscopic necrosectomy. It avoids external drains and may reduce the risk of external pancreatic fistula, although repeated sessions are often required, and complications such as bleeding or stent‐related events may occur [11].

Minimally invasive surgical necrosectomy (MIN), particularly video‐assisted retroperitoneal debridement (VARD), provides access to necrotic collections through a flank incision, often using an existing drain tract, and enables direct debridement with less physiological insult than open surgery [12]. However, minimally invasive management of necrotizing pancreatitis comprises several distinct interventions that are not interchangeable, as they differ in access route, anatomical reach, visualization, and clinical indication. These include VARD, minimally invasive retroperitoneal necrosectomy (MIRN), and retroperitoneal or transperitoneal laparoscopic debridement. In particular, retroperitoneal minimally invasive surgery differs fundamentally from endoscopic transluminal necrosectomy, which is generally selected for more central or retrogastric collections. In contrast, retroperitoneal approaches are often better suited for lateral or flank collections [13]. Open necrosectomy is now generally reserved for failure of less invasive strategies or for life‐threatening complications. Although it allows extensive debridement, it remains associated with substantial morbidity, prolonged hospital stay, and high mortality, particularly when performed early [14]. In selected cases, staged debridement (step‐up approach) may also be performed through a pre‐existing tract using rigid or flexible endoscopes, thereby avoiding formal open surgery [15].

Despite the availability of multiple therapeutic options, high‐quality comparative evidence to guide individualized selection remains limited. In particular, the relative performance of ON, MIN, and EN across short‐term and long‐term outcomes remains incompletely defined. Existing studies vary considerably in design, comparator groups, and reported endpoints, which complicates the direct interpretation of the available evidence [16, 17, 18, 19, 20].

Therefore, the present study compares these approaches in patients with necrotizing pancreatitis requiring intervention. Primary outcomes include mortality, major complications, new‐onset multiple organ failure, bleeding requiring intervention, and reintervention. Secondary outcomes include new‐onset diabetes, pancreatic exocrine insufficiency, incisional hernia, and new ICU admission. By synthesizing the currently available evidence, we aim to support evidence‐based selection of optimal interventional strategies and improve outcomes in this high‐risk population.

## 2 Methods

### 2.1 Study Design and Registration

The protocol for this systematic review was registered in the PROSPERO registry System (registration number: CRD420251128287). This systematic review and network meta‐analysis was conducted in accordance with the Preferred Reporting Items for Systematic Reviews and Meta‐Analyses extension for Network Meta‐Analyses (PRISMA‐NMA) guidelines [21]. The methodology follows the recommendations of the Cochrane Handbook for Systematic Reviews of Interventions [22].

### 2.2 Eligibility Criteria

Studies were eligible for inclusion if they: (1) were randomized controlled trials (RCTs), prospective or retrospective comparative cohort studies, or case‐matched studies; (2) enrolled adult patients (age ≥ 18 years) with confirmed necrotizing pancreatitis requiring interventional necrosectomy; (3) compared at least two of the three treatment approaches: open necrosectomy (ON), minimally invasive surgical necrosectomy (MIN, including video‐assisted retroperitoneal debridement [VARD], retroperitoneal laparoscopic debridement [RLDD], and minimally invasive retroperitoneal necrosectomy [MIRN]), or endoscopic transgastric necrosectomy (EN, including direct endoscopic necrosectomy [DEN]); and (4) reported at least one of the prespecified binary outcomes. Studies were excluded if they: (1) were single‐arm studies without a comparison group; (2) were case reports, case series (< 5 patients per arm), reviews, editorials, or meta‐analyses; (3) evaluated non‐interventional management exclusively (e.g., percutaneous drainage alone without necrosectomy); or (4) had insufficient data for effect size calculation. Single‐arm studies that met all other inclusion criteria except the comparative design were retained for a separate proportional meta‐analysis. We included studies that met these criteria, as defined by the Population, Intervention, Comparison, and Outcome (PICO) framework [23].

**Table:** 

PICO	
Population	Patients with necrotizing pancreatitis.
Intervention	Minimally invasive necrosectomy (MIN), including VARD.
Comparison	Non‐MIN techniques (open, endoscopic).
Outcome	Mortality rate, hospital stay, postoperative complications (e.g., fistulas, bleeding, wound infections, and redo surgery).

### 2.3 Information Sources and Search Strategy

A comprehensive literature search was performed in PubMed/MEDLINE, Embase, the Cochrane Central Register of Controlled Trials (CENTRAL), and Web of Science from database inception through March 2026. The search strategy combined Medical Subject Headings (MeSH) and free‐text terms including: (“necrotizing pancreatitis” OR “pancreatic necrosis” OR “infected pancreatic necrosis” OR “walled‐off necrosis”) AND (“necrosectomy” OR “debridement” OR “drainage” OR “step‐up approach”) AND (“open” OR “minimally invasive” OR “VARD” OR “retroperitoneal” OR “endoscopic” OR “transgastric” OR “laparoscopic”). Reference lists of eligible studies and relevant systematic reviews were manually screened for additional citations. No language restrictions were applied.

### 2.4 Study Selection and Data Extraction

Two independent reviewers screened titles and abstracts, followed by a full‐text review of potentially eligible articles. Disagreements were resolved by consensus. Data were extracted independently using a standardized form. All numerical data were verified through image‐based extraction directly from published manuscript tables and figures. The following variables were extracted: study characteristics (first author, year, journal, design, country, enrollment period, registration number, sample size per arm); patient demographics (age, sex, BMI, etiology, ASA class); disease severity indicators (APACHE‐II score, CT severity index, extent of necrosis, organ failure status, ICU admission, infected necrosis confirmation); and all prespecified outcomes.

### 2.5 Outcomes

For the network meta‐analysis, nine binary outcomes were analyzed: (1) mortality (in‐hospital or within follow‐up period); (2) complications (major complications composite or total complications); (3) new‐onset multiple organ failure (MOF); (4) bleeding requiring intervention; (5) new‐onset diabetes (endocrine insufficiency); (6) exocrine insufficiency (enzyme use); (7) reintervention (surgical re‐exploration for complications or persistent necrosis); (8) incisional hernia; and (9) new ICU admission post‐intervention. Outcome definitions were harmonized across studies based on the IAP/APA consensus definitions [24]. For the single‐arm proportional meta‐analysis, four outcomes were analyzed: mortality, clinical success, complications, and need for additional surgery.

### 2.6 Network Meta‐Analysis Statistical Model

To standardize the categorization of interventions by visualization, access route, and procedural intent, we applied the classification framework proposed by Loveday et al. (2011), which was developed to improve comparability and communication across multidisciplinary pancreatitis interventions [25]. A three‐node frequentist network meta‐analysis was performed using the graph‐theoretical approach [26], implemented in the netmeta package (version 3.2‐0) for R (version 4.3.3; R Foundation for Statistical Computing, Vienna, Austria). For each binary outcome, the log odds ratio (logOR) and its standard error (selogOR) were calculated for each pairwise comparison within each study. A continuity correction of 0.5 was applied to zero‐event cells. Both common‐effects and random‐effects models were fitted. The DerSimonian‐Laird estimator was used for the estimation of between‐study heterogeneity variance (tau‐squared) [27]. Open necrosectomy (ON) was designated as the reference treatment. Results are reported as odds ratios (OR) with 95% confidence intervals (CI) under the random‐effects model. The network meta‐analysis included nine comparative studies, comprising eight two‐arm studies and one three‐arm study by Bausch et al. [28]. For this multi‐arm study, which compared all three modalities simultaneously, the correlation structure among treatment effect estimates was accounted for using the multi‐arm trial adjustment within the netmeta framework.

### 2.7 Single‐Arm Proportional Meta‐Analysis

A separate proportional meta‐analysis was conducted using data from 24 single‐arm studies identified through the same systematic search. Studies were classified into two technique‐based subgroups: EN (comprising endoscopic transluminal necrosectomy [DEN/TEN] and percutaneous endoscopic necrosectomy [PEN]; 12 studies) and MIN (comprising minimal‐access retroperitoneal pancreatic necrosectomy [MIRN] and video‐assisted retroperitoneal debridement [VARD]; 11 studies). One mixed‐technique study was included in the overall pooling but excluded from subgroup comparisons. Event proportions were pooled using the logit transformation with inverse‐variance weighting under a DerSimonian‐Laird random‐effects model using the (metaprop) function. Subgroup difference tests were conducted using the *Q*‐statistic for interaction.

### 2.8 Treatment Ranking

Treatment rankings were assessed using *P*‐scores, which range from 0% (worst) to 100% (best) and have a frequentist interpretation analogous to the Bayesian SUCRA [29]. Rankings were computed under the random‐effects model with the directive “small.values = good”, as lower event rates indicate better outcomes for all endpoints.

### 2.9 Assessment of Heterogeneity and Inconsistency

Statistical heterogeneity was assessed using the generalized *Q* statistic decomposed into within‐design and between‐design components. The *I*‐squared statistic was calculated to quantify the proportion of total variability attributable to heterogeneity. Inconsistency between direct and indirect evidence was assessed using: (1) the between‐designs *Q*‐test for global inconsistency, with a significance threshold of *p* < 0.10 and (2) node‐splitting analysis, which separates direct and indirect estimates for each pairwise comparison and tests for statistically significant disagreement.

### 2.10 Subgroup Analyses

Sensitivity analyses further confirmed the robustness of the pooled estimates. Exclusion of studies judged to be at higher risk of bias, as well as leave‐one‐out analyses, did not meaningfully change effect sizes for the principal outcomes, indicating that no single study disproportionately drove the results. Collectively, these checks support the stability of our conclusions while acknowledging that residual heterogeneity likely reflects real‐world differences in patient selection, procedural expertise, and institutional step‐up protocols.

### 2.11 Risk of Bias Assessment

Risk of bias was assessed for all included studies using validated tools. For randomized controlled trials, the Cochrane Risk of Bias 2 (RoB 2) tool was used, evaluating five domains: D1 (randomization), D2 (deviations from intended interventions), D3 (missing outcome data), D4 (measurement of the outcome), and D5 (selection of the reported result). For all observational studies, including both comparative and single‐arm studies, the Risk Of Bias In Non‐randomized Studies of Interventions (ROBINS‐I) tool was used. Overall judgments were classified as Low, Some concerns/Moderate, or High/Serious risk of bias. The confidence in the NMA evidence was assessed using the CINeMA framework.

### 2.12 Publication Bias

Publication bias was assessed visually using comparison‐adjusted funnel plots for each outcome. Given the limited number of studies per comparison (< 10 in all cases), formal statistical tests were not performed due to insufficient statistical power.

### 2.13 Software

All statistical analyses were performed using R version 4.3.3. The netmeta package (version 3.2‐0) was used for network meta‐analysis, the meta package (version 8.2‐1) for proportional meta‐analysis, and ggplot2 for visualizations [30, 31, 32]. Figures were generated using cairo_pdf. Documents were produced using the officer and flextable packages.

### 2.14 Graphical Abstract and Audio Overview

A graphical abstract and an audio overview of the study were generated using NotebookLM (Google LLC, Mountain View, CA, USA; https://notebooklm.google/) to facilitate dissemination and accessibility of the findings. These materials did not influence data analysis, interpretation, or study outcomes [33]. The embedded audio file is provided as Audio 1, with a corresponding placeholder image.

**AUDIO 1:** Embedded audio overview of the study. This audio overview summarizes the main background, methods, findings, and conclusions of the network meta‐analysis. It was generated using NotebookLM and was intended only to improve accessibility and dissemination. It was not used for data extraction, analysis, interpretation, or manuscript conclusions. To view this video in the full‐text HTML version of the article, please visit https://onlinelibrary.wiley.com/doi/10.1002/wjs.70406.

## 3 Results

### 3.1 Study Selection, Characteristics, and Patient Demographics

**FIGURE 1:**
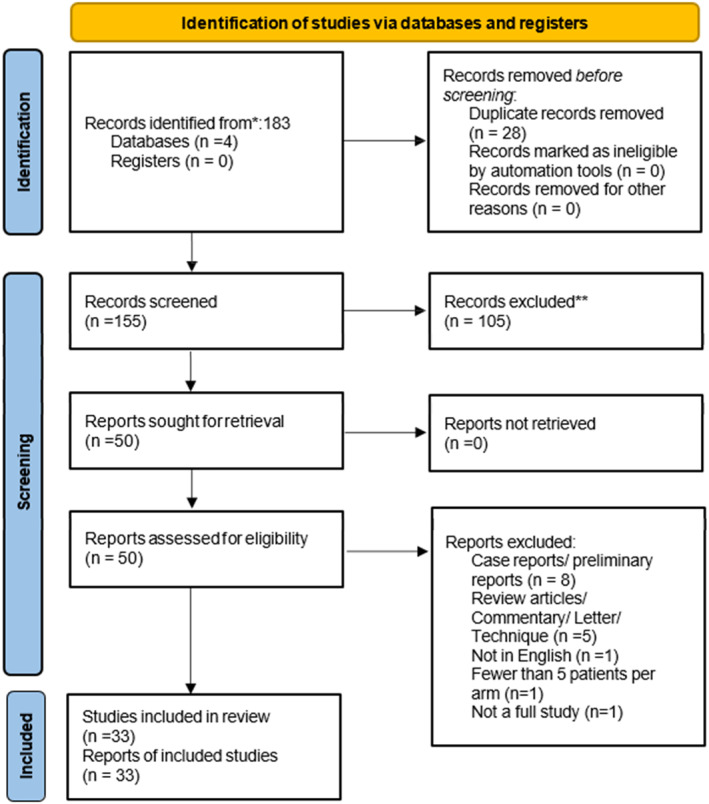
PRISMA flow diagram. *Source:* Page et al. [66].

The systematic search identified a total of 33 eligible studies, including 1786 patients. Nine comparative studies [28, 34, 35, 36, 37, 38, 39, 40, 41] were included in the network meta‐analysis: four RCTs (van Santvoort et al. [PANTER], 2010; Bakker et al. [PENGUIN], 2012; van Brunschot et al. [TENSION], 2017; Bang et al. [MISER], 2019) and five observational studies (Avudiappan et al., 2023; Bausch et al., 2012; Kumar et al., 2014; Tu et al., 2013; van Santvoort et al., 2007), enrolling a total of 560 patients across six countries. An additional 24 single‐arm studies [42, 43, 44, 45, 46, 47, 48, 49, 50, 51, 52, 53, 54, 55, 56, 57, 58, 59, 60, 61, 62, 63, 64, 65], involving 1226 patients, were included in the proportional meta‐analysis, comprising 12 EN studies (8 DEN/TEN + 4 PEN; 592 patients), 11 MIN studies (3 MARPN + 8 VARD; 490 patients), and 1 mixed‐technique study (144 patients). The majority of studies were retrospective (27/33; 81.8%), with four RCTs and one prospective single‐arm study. Studies spanned 14 countries across four continents, with enrollment periods from 1995 to 2023. Across all studies, the mean/median age ranged from 27 to 64 years, and male sex predominated (50%–91%). The most common etiologies were gallstones (25%–92%) and alcohol (7%–77%). Disease severity was generally comparable within studies: APACHE‐II scores ranged from 5.7 to 33.7, CT severity indices from 5.3 to 10, and baseline multiple organ failure rates from 0% to 73%. Infected necrosis was confirmed in 72%–100% of patients. The complete breakdown of study characteristics and baseline demographics is presented in Table 1A (comparative studies) and Table 1B (single‐arm studies). The Prisma flow diagram is presented in Figure 1.

**TABLE 1A:** Characteristics and baseline demographics of all included studies: Comparative studies (network meta‐analysis).

Study	Journal	Design	Country	Comparison	*N*	Age	Male (%)	Etiology	APACHE‐II	MOF (%)
van Santvoort et al. [34]	*NEJM*	MC RCT	NL	ON versus MIN	88	57.6/57.4	72/73	GS 60/64%	14.6/15.0	35/29%
Bakker et al. [35]	*JAMA*	MC RCT	NL	MIN versus EN	20	64/62	80/60	GS 70/60%	11/10	10/20%
van Brunschot et al. [36]	*Lancet*	MC RCT	NL	MIN versus EN	98	63/60	67/62	GS 51/64%	9/10	18/15%
Bang et al. [37]	*Gastroenterology*	SC RCT	USA	MIN versus EN	66	55.6/52.9	65/66	GS 41/25%	30/21	21/22%
Avudiappan et al. [38]	*Surg Open Sci*	Retro	India	ON versus MIN	122	37/35.5	83/80	Alc 56/43%	10.8/11.1	53/41%
Bausch et al. [28]	*Surgery*	Retro	Germany	ON versus MIN versus EN	62	64/61/58	57/79/56	Alc 17/21/22%	NR	73/14/0%
Kumar et al. [39]	*Pancreas*	Matched	USA	MIN versus EN	24	58.9/53.3	67/75	GS 58/42%	10.1/9.4	0/0%
Tu et al. [40]	*Surg Endosc*	Retro	China	ON versus MIN	50	51.3/48.7	56/59	GS 83/88%	13.3/13.4	NR
van Santvoort et al. [41]	*World J Surg*	Case‐match	NL	ON versus MIN	30	52/53	80/67	GS 53/33%	9/9	27/27%

*Note:* Values per arm (Arm 1/Arm 2/Arm 3).

Abbreviations: EN = Endoscopic, MC = Multicenter, MIN = Minimally Invasive Necrosectomy, NL = Netherlands, ON = Open Necrosectomy, SC = Single‐center.

**TABLE 1B:** Characteristics and baseline demographics of all included studies: Single‐arm studies (proportional meta‐analysis).

Study	Technique	Subgroup	Design	Country	*N*	Age	Male (%)	GS (%)	Alc (%)	APACHE‐II	BMI
Yan et al. [42]	EN	EN	Retro	China	82	50.7 ± 14.3	61.0	50.0	12.2	12.2	NR
Garg et al. [43]	EN	EN	Retro	India	57	35.6 ± 13.3	73.7	29.8	42.1	NR	NR
Kumta et al. [44]	EN	EN	Retro	USA	80	54.5 ± 15.1	60.0	41.3	22.5	NR	NR
Oblizajek et al. [45]	EN	EN	Retro	USA	103	56 ± 16	55.3	40.8	21.4	11	NR
Guo et al. [46]	EN	EN	Retro	China	34	46.1 ± 12.8	64.7	44.1	17.6	12.8	NR
Jain et al. [47]	EN	EN	Retro	India	91	37.8 ± 13.2	74.7	30.8	41.8	NR	NR
Al‐Efishat et al. [48]	EN	EN	Retro	Multi	120	55.3 ± 15.8	56.7	43.3	20.8	NR	NR
Takenaka et al. [49]	EN	EN	Retro	Japan	48	56.8 ± 14.2	68.8	35.4	29.2	NR	NR
Dhingra et al. [50]	PEN	EN	Retro	Australia	15	NR (18–85)	66.7	NR	NR	NR	NR
Saumoy et al. [51]	PEN	EN	Retro	USA	9	NR	77.8	NR	NR	NR	NR
Mangiafico et al. [52]	PEN	EN	Retro	Italy	18	60 ± 12	50.0	38.9	33.3	NR	20–32
Tenorio‐Gonzalez et al. [53]	PEN	EN	Retro	France	15	59 ± 12.7	80.0	60.0	33.3	NR	21 (17–26)
Liu et al. [54]	MARPN	MIN	Retro	China	164	48 (37–57)	71.3	57.3	18.3	11 (8–15)	NR
Wan et al. [55]	MARPN	MIN	Retro	China	73	48.3 ± 12.5	71.2	45.2	19.2	13.6	NR
Li et al. [56]	MARPN	MIN	Retro	China	54	51.2 ± 3.1	72.2	64.8	11.1	12.4	NR
Wu et al. [57]	VARD	MIN	Retro	USA	10	41 ± 15	50.0	40.0	20.0	19 (10–29)	34 (21–51)
Budkule et al. [58]	VARD	MIN	Retro	India	22	48.6 (39–68)	72.7	31.8	36.4	NR	27.8
Chaves et al. [59]	VARD	MIN	Retro	Colombia	12	55.9 ± 13.7	75.0	91.7	8.3	NR	NR
Garcia‐Urena et al. [60]	VARD	MIN	Retro	Spain	7	44 (31–63)	85.7	NR	NR	5.7	NR
Eickhoff et al. [61]	VARD	MIN	Retro	Germany	9	53 (28–72)	66.7	NR	NR	15.2	30
Ulagendra Perumal et al. [62]	VARD	MIN	Retro	India	26	38.6 ± 9.9	88.5	26.9	69.2	7.7	NR
Wei et al. [63]	VARD	MIN	Prosp	China	21	42.9 ± 11.7	52.4	28.6	19.0	8.7	22.8
Sileikis et al. [64]	VARD	MIN	Retro	Lithuania	13	42.8 ± 9.2	NR	NR	76.9	NR	NR
Ebrahim et al. [65]	MIXED	MIXED	Retro	Denmark	144	60 (49–69)	54.9	52.0	17.0	NR	NR

Abbreviations: Alc = Alcohol, EN = endoscopic necrosectomy (DEN/TEN), EN subgroup = EN + PEN, GS = Gallstones, MARPN = minimal‐access retroperitoneal pancreatic necrosectomy, MIN subgroup = MARPN + VARD, NR = Not Reported, PEN = percutaneous endoscopic necrosectomy, Prosp = Prospective, Retro = Retrospective, VARD = video‐assisted retroperitoneal debridement.

### 3.2 Network Structure

The network comprised three treatment nodes (ON, MIN, EN) connected by three types of direct comparisons: ON versus MIN (4 studies), MIN versus EN (4 studies), and the three‐arm Bausch et al. study contributing to all comparisons. This formed a well‐connected closed‐loop triangular network. Contributing studies ranged from 3 to 9 per outcome.

### 3.3 Network Meta‐Analysis Outcomes

**FIGURE 2:**
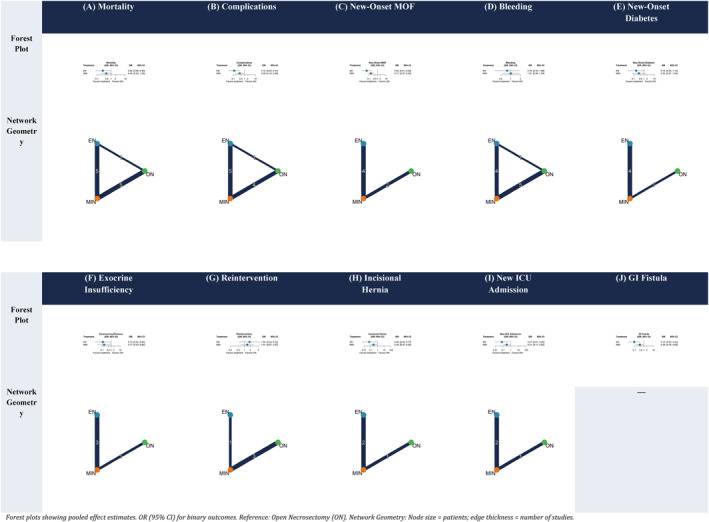
Network meta‐analysis: Forest plots and network geometry.

The complete NMA results for all outcomes are summarized in Table 2 and visualized in Figure 2.

**TABLE 2:** Network meta‐analysis: Summary of all outcomes (random‐effects OR [95% CI]).

Outcome	Studies	EN versus ON	MIN versus ON	MIN versus EN	*I* ^2^ (%)
Mortality	9	0.23 [0.06–0.91]	0.41 [0.16–1.05]	N/A	48.7
Complications	8	0.11 [0.03–0.4]	0.37 [0.14–1.01]	N/A	59.6
New onset MOF	6	0.05 [0.01–0.19]	0.15 [0.06–0.4]	N/A	0
Bleeding	8	0.74 [0.3–1.84]	0.96 [0.51–1.81]	N/A	0
New onset diabetes	5	0.17 [0.03–1.12]	0.32 [0.07–1.55]	N/A	38.9
Exocrine insufficiency	4	0.1 [0.01–0.81]	0.15 [0.03–0.84]	N/A	29.4
Reintervention	4	1.86 [0.53–6.5]	1.41 [0.65–3.06]	N/A	0
Incisional hernia	3	0.05 [0–0.72]	0.23 [0.06–0.9]	N/A	0
New ICU admission	3	0.06 [0.01–0.57]	0.29 [0.11–0.8]	N/A	0

*Note:* OR < 1 favors the first treatment.

#### 3.3.1 Mortality

All nine studies reported mortality data (Figure 2A). Under the random‐effects model, EN was significantly superior to ON (OR 0.24, 95% CI 0.06–0.95, *p* = 0.04). MIN showed a strong trend toward superiority over ON that approached statistical significance (OR 0.41, 95% CI 0.16–1.04, *p* = 0.06). No significant difference was found between MIN and EN (OR 1.72, 95% CI 0.56–5.27, *p* = 0.35). Heterogeneity was moderate (*I*‐squared = 48%, *Q* = 15.38, *df* = 8, *p* = 0.052; Table 3). EN had the highest *P*‐score (90.4%), followed by MIN (57.0%) and ON (2.6%).

**TABLE 3:** Heterogeneity and inconsistency tests.

Outcome	*Q*	*df*	*p*‐value	*I* ^2^ (%)	tau^2^
Mortality	15.58	8	0.0488	48.7	0.6120
Bleeding	4.59	7	0.7099	0	0.0000
Complications	17.33	7	0.0154	59.6	0.6692
New Onset MOF	2.67	4	0.6142	0	0.0000
New Onset diabetes	4.91	3	0.1787	38.9	0.3818
Exocrine insufficiency	2.83	2	0.2428	29.4	0.3126
Reintervention	0.87	2	0.6460	0	0.0000
Incisional hernia	0.05	1	0.8150	0	0.0000
New ICU admission	0.82	1	0.3651	0	0.0000

*Note:* *p* < 0.10 indicates significant heterogeneity.

#### 3.3.2 Complications

Eight studies contributed data on complications (Figure 2B). EN was significantly superior to ON (OR 0.11, 95% CI 0.03–0.40, *p* < 0.001) and also superior to MIN (OR for MIN vs. EN: 3.41, 95% CI 1.31–8.92, *p* = 0.01). MIN showed a trend toward superiority over ON (OR 0.37, 95% CI 0.14–1.01, *p* = 0.053). Heterogeneity was substantial (*I*‐squared = 60%, *Q* = 17.33, *df* = 7, *p* = 0.015; Table 3). EN *P*‐score: 99.7%.

#### 3.3.3 New‐Onset Multiple Organ Failure

Six studies provided data (Figure 2C). Both EN and MIN were significantly superior to ON: EN versus ON (OR 0.05, 95% CI 0.01–0.19, *p* < 0.001) and MIN versus ON (OR 0.15, 95% CI 0.06–0.40, *p* < 0.001). EN was also superior to MIN (OR 3.18, 95% CI 1.07–9.45, *p* = 0.04). *I*‐squared = 0%; Table 3. EN *P*‐score: 99.1%.

#### 3.3.4 Bleeding Requiring Intervention

Eight studies reported bleeding data (Figure 2D). No significant differences were observed between any treatment pair: EN versus ON (OR 0.75, 95% CI 0.30–1.86, *p* = 0.54), MIN versus ON (OR 0.96, 95% CI 0.51–1.81, *p* = 0.91), and MIN versus EN (OR 1.28, 95% CI 0.60–2.77, *p* = 0.52). *I*‐squared = 0%; Table 3.

#### 3.3.5 New‐Onset Diabetes

Five studies reported data (Figure 2E). EN showed a trend toward superiority over ON (OR 0.18, 95% CI 0.03–1.04, *p* = 0.055). MIN versus ON was non‐significant (OR 0.32, 95% CI 0.07–1.41, *p* = 0.13). *I*‐squared = 34%. EN *P*‐score: 92.8%.

#### 3.3.6 Exocrine Insufficiency

Four studies contributed data (Figure 2F). Both EN and MIN were significantly superior to ON: EN versus ON (OR 0.12, 95% CI 0.02–0.72, *p* = 0.02) and MIN versus ON (OR 0.15, 95% CI 0.03–0.71, *p* = 0.02). No significant difference between EN and MIN (OR 1.30, 95% CI 0.49–3.40, *p* = 0.60). *I*‐squared = 19%.

#### 3.3.7 Reintervention

Four studies reported data (Figure 2G). No significant differences between any pair. *I*‐squared = 0%. ON had the highest *P*‐score (82.0%), whereas EN ranked lowest (23.0%; Table 3). This was the only outcome where ON ranked best, reflecting the more radical initial debridement achieved with open surgery.

#### 3.3.8 Incisional Hernia

Three studies reported data (Figure 2H). MIN was significantly superior to ON (OR 0.23, 95% CI 0.06–0.90, *p* = 0.03). EN showed a trend toward superiority over ON (OR 0.07, 95% CI 0.00–1.05, *p* = 0.05). *I*‐squared = 0%. EN *P*‐score: 90.6%.

#### 3.3.9 New ICU Admission

Three studies reported data (Figure 2I; Supporting Information S2: Table S1). Both EN and MIN were significantly superior to ON: EN versus ON (OR 0.06, 95% CI 0.01–0.57, *p* = 0.01) and MIN versus ON (OR 0.29, 95% CI 0.11–0.80, *p* = 0.02). *I*‐squared = 0%. EN *P*‐score: 96.6%.

#### 3.3.10 Gastrointestinal Fistula

Three studies were included (Figure 2J; Supporting Information S2: Table S2). EN and MIN both showed significantly lower fistula rates compared with ON (EN: OR 0.06; MIN: OR 0.29). No difference was observed between EN and MIN. Heterogeneity was negligible (*I* ^2^ = 0%), with EN ranking highest (*P*‐score 96.6%).

### 3.4 Consistency and Heterogeneity

The between‐designs *Q*‐test did not detect significant global inconsistency for any NMA outcome (all *p* > 0.05), confirming agreement between direct and indirect evidence (Table 3). Node‐splitting analysis demonstrated no statistically significant differences between direct and indirect estimates for any comparison. The majority of NMA outcomes had *I*‐squared = 0%; substantial heterogeneity was observed for Complications (*I*‐squared = 60%) and moderate heterogeneity for Mortality (*I*‐squared = 48%). Funnel plots showed no major asymmetry.

### 3.5 Treatment Rankings

Overall treatment ranking by *P*‐scores is presented in Table 4. EN consistently ranked as the best treatment in 8 of 9 outcomes, with *P*‐scores ranging from 73.5% (Bleeding) to 99.7% (Complications). MIN consistently ranked second. ON ranked last in 8 of 9 outcomes. The sole exception was Reintervention, where ON ranked first (*P*‐score 82.0%).

**TABLE 4:** Treatment rankings (*P*‐scores).

Outcome	ON (%)	MIN (%)	EN (%)
Mortality	2.5	56	91.5
Bleeding	35.8	39.8	74.5
Complications	1.3	49	99.7
New onset MOF	0	50.7	99.3
New onset diabetes	5.6	51.7	92.7
Exocrine insufficiency	1.5	62.3	86.2
Reintervention	82	45	23
Incisional hernia	1.5	54	94.5
New ICU admission	0.8	52.7	96.6

*Note:* Higher *P*‐score = better ranking (0%–100%).

### 3.6 Risk of Bias Assessment

Risk of bias was assessed for all 33 included studies (Tables 5A and 5B). The four RCTs (PANTER, PENGUIN, TENSION, MISER) were assessed as having an overall Low risk of bias using RoB 2. All demonstrated adequate randomization, low rates of missing data, and appropriate outcome measurement; some concerns were noted for deviations from intended interventions (D2) as blinding was not feasible. Among the five comparative observational studies, ROBINS‐I assessment classified four as Moderate risk (Avudiappan et al. [38]; Bausch et al. [28]; Kumar et al. [39]; Tu et al. [40]) and one as Low risk (van Santvoort et al. [41]), which used rigorous case‐matching (Table 5A).

**TABLE 5A:** Risk of bias assessment (NMA).

Study	Tool	D1	D2	D3	D4	D5	Overall
van Santvoort et al. [34]	RoB 2	Low	Some	Low	Low	Low	Low
Bakker et al. [35]	RoB 2	Low	Low	Low	Low	Low	Low
van Brunschot et al. [36]	RoB 2	Low	Some	Low	Low	Low	Low
Bang et al. [37]	RoB 2	Low	Some	Low	Low	Low	Low
Avudiappan et al. [38]	ROBINS‐I	N/A	Moderate	Low	Moderate	Moderate	Moderate
Bausch et al. [28]	ROBINS‐I	N/A	Moderate	Low	Moderate	Moderate	Moderate
Kumar et al. [39]	ROBINS‐I	N/A	Moderate	Low	Moderate	Moderate	Moderate
Tu et al. [40]	ROBINS‐I	N/A	Moderate	Low	Moderate	Moderate	Moderate
van Santvoort et al. [41]	ROBINS‐I	N/A	Low	Low	Moderate	Low	Low

*Note:* D1–D5 = domains per tool, RCTs: RoB 2; Observational: ROBINS‐I.

**TABLE 5B:** Risk of bias assessment—Single‐arm studies.

Study	Type	Tool	Overall
Yan et al. [42]	SA‐EN	ROBINS‐I	Moderate
Garg et al. [43]	SA‐EN	ROBINS‐I	Moderate
Kumta et al. [44]	SA‐EN	ROBINS‐I	Moderate
Oblizajek et al. [45]	SA‐EN	ROBINS‐I	Moderate
Guo et al. [46]	SA‐EN	ROBINS‐I	Moderate
Jain et al. [47]	SA‐EN	ROBINS‐I	Moderate
Al‐Efishat et al. [48]	SA‐EN	ROBINS‐I	Moderate
Takenaka et al. [49]	SA‐EN	ROBINS‐I	Moderate
Dhingra et al. [50]	SA‐PEN	ROBINS‐I	Moderate
Saumoy et al. [51]	SA‐PEN	ROBINS‐I	Moderate
Mangiafico et al. [52]	SA‐PEN	ROBINS‐I	Low
Tenorio‐Gonzalez et al. [53]	SA‐PEN	ROBINS‐I	Moderate
Liu et al. [54]	SA‐MARPN	ROBINS‐I	Moderate
Wan et al. [55]	SA‐MARPN	ROBINS‐I	Moderate
Li et al. [56]	SA‐MARPN	ROBINS‐I	Moderate
Wu et al. [57]	SA‐VARD	ROBINS‐I	Moderate
Budkule et al. [58]	SA‐VARD	ROBINS‐I	Moderate
Chaves et al. [59]	SA‐VARD	ROBINS‐I	Moderate
Garcia‐Urena et al. [60]	SA‐VARD	ROBINS‐I	Moderate
Eickhoff et al. [61]	SA‐VARD	ROBINS‐I	Moderate
Ulagendra Perumal et al. [62]	SA‐VARD	ROBINS‐I	Moderate
Wei et al. [63]	SA‐VARD	ROBINS‐I	Low
Sileikis et al. [64]	SA‐VARD	ROBINS‐I	Moderate
Ebrahim et al. [65]	SA‐MIXED	ROBINS‐I	Moderate

Abbreviations: NMA = Network Meta‐Analysis studies, RoB 2 = Cochrane Risk of Bias 2 (RCTs), ROBINS‐I = Risk Of Bias In Non‐randomized Studies of Interventions (observational/single‐arm), SA = Single‐Arm studies.

The 24 single‐arm studies (Table 5B) were assessed using ROBINS‐I adapted for single‐arm designs. The majority were rated as Moderate risk of bias due to retrospective design, potential selection bias from tertiary referral center recruitment, and inconsistencies in outcome definitions. No study was assessed as having a serious or critical risk of bias. The CINeMA confidence assessment (Supporting Information S2: Table S3) classified overall evidence as Moderate for 2 NMA outcomes (New‐Onset MOF, Bleeding) and Low for 7 outcomes, primarily driven by imprecision and heterogeneity.

### 3.7 Single‐Arm Proportional Meta‐Analysis Outcomes

**FIGURE 3:**
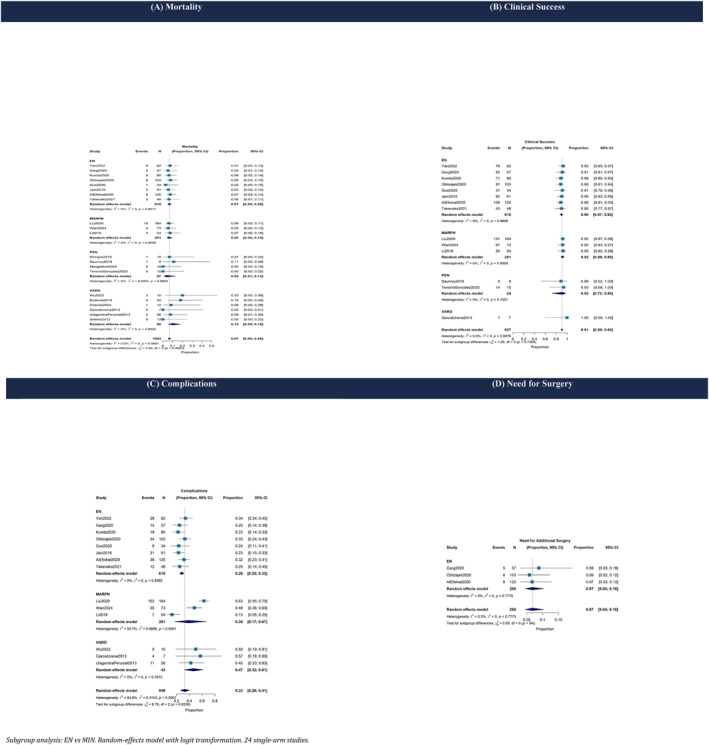
Single‐arm proportional meta‐analysis. Subgroup analysis: EN versus MIN. Random‐effects model with logit transformation. Twenty four single‐arm studies.

The proportional meta‐analysis results with subgroup difference tests are summarized in Tables 6 and 7 and Figure 3 and Supporting Information S1: Figures S1–S4. The *P*‐Score Bar Cart is shown in Supporting Information S1: Figure S5, and the Rankogram is demonstrated in Supporting Information S1: Figure S6.

**TABLE 6:** Outcomes of included single‐arm studies.

Study	Subgroup	*N*	Mort (*n*)	Mort (%)	CS (*n*)	CS (%)	Comp (*n*)	Comp (%)
Yan et al. [42]	EN	82	6	7.3	76	92.7	28	34.1
Garg et al. [43]	EN	57	3	5.3	52	91.2	14	24.6
Kumta et al. [44]	EN	80	5	6.2	71	88.8	18	22.5
Oblizajek et al. [45]	EN	103	8	7.8	91	88.3	34	33.0
Guo et al. [46]	EN	34	1	2.9	31	91.2	8	23.5
Jain et al. [47]	EN	91	5	5.5	82	90.1	21	23.1
Al‐Efishat et al. [48]	EN	120	9	7.5	106	88.3	38	31.7
Takenaka et al. [49]	EN	48	3	6.2	43	89.6	12	25.0
Dhingra et al. [50]	EN	15	1	6.7	14	93.3	2	13.3
Saumoy et al. [51]	EN	9	1	11.1	8	88.9	0	0.0
Mangiafico et al. [52]	EN	18	0	0.0	17	94.4	5	27.8
Tenorio‐Gonzalez et al. [53]	EN	15	0	0.0	14	93.3	4	26.7
Liu et al. [54]	MIN	164	10	6.1	151	92.1	103	62.8
Wan et al. [55]	MIN	73	4	5.5	67	91.8	35	47.9
Li et al. [56]	MIN	54	4	7.4	50	92.6	7	13.0
Wu et al. [57]	MIN	10	2	20.0	NR	NR	5	50.0
Budkule et al. [58]	MIN	22	4	18.2	NR	NR	NR	NR
Chaves et al. [59]	MIN	12	1	8.3	NR	NR	5	41.7
Garcia‐Urena et al. [60]	MIN	7	0	0.0	7	100.0	4	57.1
Eickhoff et al. [61]	MIN	9	0	0.0	NR	NR	0	0.0
Ulagendra Perumal et al. [62]	MIN	26	2	7.7	NR	NR	11	42.3
Wei et al. [63]	MIN	21	1	4.8	14	66.7	6	28.6
Sileikis et al. [64]	MIN	13	0	0.0	NR	NR	0	0.0
Ebrahim et al. [65]	MIXED	144	24	16.7	120	83.3	10	6.9

Abbreviations: Comp = Complications, CS = Clinical Success, Mort = Mortality, NR = Not Reported.

**TABLE 7:** Subgroup difference tests: EN versus MIN (proportional meta‐analysis).

Outcome	Overall (%)	95% CI	*I* ^2^ (%)	Tau^2^ (%)	EN (%)	MIN (%)	*p*‐value
Mortality	7.0	(5.6%–8.7%)	0.0	0.0000	6.6	7.7	0.5011
Clinical success	89.8	(87.7%–91.6%)	0.0	0.0000	89.9	88.9	0.7978
Complications	30.2	(23.7%–37.6%)	78.9	0.4046	27.9	36.6	0.2241
Need for surgery	7.1	(5.2%–9.6%)	0.0	0.0000	6.3	9.0	0.3900

*Note:* Pooled using logit‐transformed proportional meta‐analysis with DerSimonian–Laird random‐effects model.

Abbreviations: EN subgroup = EN + PEN (12 studies), MIN subgroup = MARPN + VARD (11 studies).

#### 3.7.1 Mortality (Single‐Arm)

The pooled mortality was 7.0% (95% CI: 5.6%–8.7%; *I*‐squared = 0%). EN subgroup: 6.6% versus MIN subgroup: 7.7%. Subgroup difference: *P* = 0.501 (Figure 3A).

#### 3.7.2 Clinical Success (Single‐Arm)

The pooled clinical success rate was 89.8% (95% CI: 87.7%–91.6%; *I*‐squared = 0%). EN: 89.9% versus MIN: 88.9%. Subgroup difference: *P* = 0.798 (Figure 3B).

#### 3.7.3 Complications (Single‐Arm)

The pooled complication rate was 30.2% (95% CI: 23.7%–37.6%; *I*‐squared = 78.9%). EN: 27.9% versus MIN: 36.6%. Subgroup difference: *P* = 0.224 (Figure 3C). The high heterogeneity reflects differences in complication definitions and disease severity.

#### 3.7.4 Need for Additional Surgery (Single‐Arm)

The pooled rate was 7.1% (95% CI: 5.2%–9.6%; *I*‐squared = 0%). EN: 6.3% versus MIN: 9.0%. Subgroup difference: *P* = 0.390 (Figure 3D).

#### 3.7.5 Summary of Single‐Arm Findings

No statistically significant differences were found between the EN and MIN subgroups for any outcome (Table 7). Both approaches demonstrated comparable mortality (6.6% vs. 7.7%), clinical success (89.9% vs. 88.9%), complication rates (27.9% vs. 36.6%), and need for surgery (6.3% vs. 9.0%). These findings corroborate the NMA results from comparative studies.

## 4 Discussion

The present systematic review, network meta‐analysis, and proportional meta‐analysis was designed to compare open necrosectomy (ON), minimally invasive surgical necrosectomy (MIN), and endoscopic transgastric necrosectomy (EN) in adults with necrotizing pancreatitis requiring intervention. Specifically, we aimed to evaluate peri‐interventional and clinically relevant postoperative outcomes, including mortality, complications, organ failure, bleeding, pancreatic functional sequelae, reinterventions, incisional hernia, and ICU‐related outcomes, while also integrating evidence from non‐comparative cohorts through proportional meta‐analysis. To our knowledge, this is the first study to combine a three‐node comparative network meta‐analysis with a parallel proportional meta‐analysis on this topic, thereby allowing both relative treatment ranking and technique‐specific event estimation across a broader evidence base.

Our findings indicate that EN achieved the most favorable overall performance across the network. It ranked best in the large majority of analyzed outcomes and demonstrated significant advantages over ON in mortality, overall complications, new‐onset multiple organ failure, exocrine insufficiency, and new ICU admission. In addition, EN was superior to MIN for overall complications and new‐onset multiple organ failure. MIN also performed better than ON for several key endpoints, particularly new‐onset multiple organ failure, exocrine insufficiency, incisional hernia, and ICU admission. By contrast, ON ranked best only for reintervention, likely reflecting the greater radicality of primary debridement during an upfront open procedure. The separate single‐arm proportional meta‐analysis complemented these findings by showing high clinical success and low mortality overall, with no statistically significant differences between EN and MIN subgroups.

Mortality was one of the most clinically relevant findings of this analysis. EN was associated with significantly lower mortality than ON, whereas MIN showed a near‐significant trend in the same direction. This result is biologically plausible and clinically coherent. Endoscopic transluminal approaches avoid laparotomy, minimize contamination of the peritoneal cavity, reduce surgical trauma, and may attenuate the systemic inflammatory response in a critically ill population already vulnerable to sepsis and organ dysfunction [37].

The PENGUIN trial by Bakker et al. [35] showed that endoscopic necrosectomy reduced the inflammatory response and improved the composite clinical endpoint compared with surgical necrosectomy, supporting the concept that less invasive transluminal therapy may translate into better systemic outcomes. In parallel, the PANTER trial by van Santvoort et al. [34] demonstrated that a step‐up strategy was superior to open necrosectomy for major clinical outcomes, thereby indirectly supporting the mortality disadvantage of more invasive open surgery. However, the TENSION trial by van Brunschot et al. [36] did not demonstrate superiority of the endoscopic over the surgical step‐up approach for the composite of death and major complications, suggesting that mortality differences may depend on patient selection, anatomy of necrosis, and the extent to which modern minimally invasive surgery is used instead of classical open necrosectomy. Taken together, our findings reinforce the notion that the main mortality penalty lies with ON, whereas EN appears to provide the most favorable balance between adequacy of source control and physiologic preservation.

The reduction in overall complications with EN is another central finding. EN was superior to both ON and MIN, whereas MIN only approached superiority over ON. This pattern is highly consistent with the modern step‐up philosophy and with the MISER trial by Bang et al. [37], which directly demonstrated lower major complications and lower costs with an endoscopic transluminal approach compared with minimally invasive surgery. Likewise, the randomized‐trials meta‐analysis by Bang et al. [67] concluded that endoscopic interventions were superior to minimally invasive surgical approaches for infected necrotizing pancreatitis, especially for major procedure‐related morbidity. Conversely, the ExTENSION follow‐up of the TENSION trial by Onnekink et al. [68] showed that long‐term superiority of endoscopy over surgery was less pronounced for broad composite outcomes, reminding us that short‐term complication advantages may not always persist uniformly during extended follow‐up. The most likely interpretation is that EN reduces access‐related morbidity, postoperative fistula, and procedure‐driven physiologic stress. Although MIN preserves many advantages over ON, it remains more invasive than transluminal endoscopic necrosectomy. Importantly, both approaches (EN and MIN) should not be performed simultaneously, as combining them may increase the risk of fistula formation and consequently severe complications. Conceptually, MIN is primarily suited for laterally located necrosis, whereas EN is better adapted for centrally located collections [37, 69].

New‐onset multiple organ failure emerged as one of the clearest discriminators between techniques. Both EN and MIN were superior to ON, and EN also outperformed MIN. This is especially important because organ failure is the dominant mediator of death in severe necrotizing pancreatitis. The TENSION trial by van Brunschot et al. [36] and the MISER trial by Bang et al. [37] both support the principle that endoscopic step‐up management can reduce procedure‐related physiologic deterioration compared with more invasive surgical strategies. Similarly, Haney et al. 2020 [70], reported in a systematic review of randomized trials that endoscopic treatment significantly reduced new‐onset organ failure compared with surgery. A possible counterpoint is that the PANTER trial by van Santvoort et al. [34] established that even a surgical step‐up strategy can markedly reduce major morbidity relative to ON, indicating that the detrimental effect is not surgery per se, but the invasiveness and timing of the intervention. Therefore, our data suggest a graded hierarchy: ON appears worst, MIN improves upon ON, and EN offers the strongest protection against post‐interventional organ failure, likely because it combines source control with the least systemic insult.

Exocrine insufficiency was significantly less frequent after both EN and MIN than after ON, whereas EN and MIN did not differ significantly from one another. Preservation of pancreatic function is clinically meaningful because many survivors of necrotizing pancreatitis face prolonged nutritional compromise and pancreatic enzyme dependence [71]. The advantage of less invasive modalities likely reflects reduced collateral pancreatic and peripancreatic tissue injury, less devascularization, and less disruption of residual viable parenchyma, as per the American Gastroenterological Association clinical practice update [72, 73].

Long‐term follow‐up from ExTENSION by Onnekink et al. [68] addressed pancreatic insufficiency after endoscopic and surgical step‐up strategies, supporting continued attention to functional outcomes beyond early postoperative events. More broadly, the study‐level meta‐analysis by Hollemans et al. on pancreatic exocrine insufficiency after acute pancreatitis [14], highlighted the substantial burden of long‐term exocrine dysfunction after severe pancreatic injury. On the other hand, the lack of a significant difference between EN and MIN in our network indicates that once open surgery is avoided, preservation of exocrine function may depend more on the underlying extent of necrosis than on whether the access route is transluminal or retroperitoneal. Thus, the main clinical message is that organ‐preserving strategies matter, and ON may exact the highest long‐term functional cost.

Incisional hernia was less frequent after MIN compared with ON. This is consistent with the technical nature of the procedures, as ON is associated with a higher risk of fascial failure in patients exposed to sepsis, malnutrition, repeated interventions, and impaired wound healing, whereas MIN minimizes abdominal wall trauma [74].

New ICU admissions were significantly lower after both EN and MIN compared with ON. This endpoint likely integrates several adverse pathways, including procedure‐related systemic stress, respiratory compromise, sepsis escalation, and management of postoperative complications. The PANTER and MISER trials support this interpretation, showing reduced severe morbidity with step‐up approaches. However, TENSION and ExTENSION indicate that endoscopy does not consistently outperform surgery in long‐term outcomes, suggesting ICU benefits are mainly driven by early postoperative effects. Nonetheless, fewer ICU readmissions favor less invasive strategies [75, 76].

Several outcomes did not differ significantly across treatment arms. Bleeding requiring intervention was comparable among EN, MIN, and ON, indicating that hemorrhage remains a shared hazard of debridement in highly inflamed and vascularized necrotic fields regardless of access route. Re‐intervention did not differ significantly, although ON ranked highest, likely because more aggressive upfront clearance may reduce the need for subsequent procedures in selected patients, or because early mortality may preclude the opportunity for re‐intervention.

New‐onset diabetes showed only a non‐significant trend favoring EN over ON, suggesting a possible endocrine preservation effect that current comparative evidence remains underpowered to confirm. Importantly, the single‐arm proportional meta‐analysis also showed no statistically significant subgroup differences between EN and MIN for mortality, clinical success, complications, or need for additional surgery, thereby supporting the view that both minimally invasive paradigms are effective and that the main inferiority lies with ON rather than a major absolute separation between EN and MIN in every endpoint.

### 4.1 Clinical Implications

From a practical perspective, these findings suggest a paradigm shift in the management of necrotizing pancreatitis. Open necrosectomy should no longer be considered a default approach but rather reserved for rescue scenarios, including failure of less invasive strategies, inaccessible necrosis, or life‐threatening complications. Endoscopic necrosectomy emerges as the preferred first‐line option when anatomical conditions allow transluminal access and sufficient expertise is available, particularly for centrally located or retrogastric collections. Minimally invasive surgical approaches remain essential, especially for laterally distributed necrosis or when endoscopic options are limited. Overall, these results support a tailored multidisciplinary strategy in which treatment selection is guided by anatomical distribution, timing, institutional expertise, and the patient's physiological status.

### 4.2 Strengths Limitations

This study has several strengths. It adhered to PRISMA‐NMA methodology and Cochrane principles, registered in Prospero, incorporated both randomized and observational comparative evidence, and complemented the network analysis with a proportional meta‐analysis of single‐arm studies to broaden external validity. The three‐node closed‐loop network enabled direct and indirect comparison among ON, MIN, and EN, and treatment ranking by *P*‐scores provided a clinically intuitive hierarchy across outcomes. Risk of bias was assessed with validated instruments; overall inconsistency was not detected, and most outcomes demonstrated low heterogeneity, strengthening confidence in the overall direction of the findings.

Nevertheless, the limitations should be acknowledged. Most included studies were retrospective, and although no study was rated as having serious or critical risk of bias, residual confounding and treatment‐selection bias remain unavoidable. Definitions of complications and thresholds for intervention were not fully uniform across cohorts, and substantial heterogeneity was present for some outcomes, particularly overall complications and mortality. The comparative network was based on only nine studies, with relatively sparse data for some endpoints, such as incisional hernia and ICU admission. In addition, MIN was a composite category that included VARD, RLDD, and MIRN, whereas EN also encompassed related transluminal techniques; this grouping was methodologically necessary but may mask important within‐category differences.

Another important point: the outcomes are influenced by necrosis distribution, timing, comorbidities, and institutional expertise, with treatment allocation often dictated by available modalities rather than randomization, leading to crossovers. Subgroup analyses were also limited by small sample sizes, low event rates, and clinical and methodological heterogeneity.

Most reports lacked detailed data on necrosis site, burden, and baseline patient condition, whereas surgical expertise and standardization were inconsistently described, restricting generalizability. Many studies did not report critical disease‐level modifiers, including necrosis composition (predominantly liquid vs. solid/organized) and the primary indication for intervention (infected necrosis vs. symptomatic sterile necrosis, such as pressure effects).

Finally, outcome reporting on long‐term endocrine and exocrine function remained limited, and confidence in several network estimates was low because of imprecision. These constraints mean that the present findings should guide, but not oversimplify, individualized decision‐making.

## 5 Conclusion

Our study suggests that endoscopic necrosectomy offers the most favorable overall outcome profile for patients with necrotizing pancreatitis requiring intervention. Minimally invasive surgical approaches also outperform open necrosectomy for several important short‐ and long‐term outcomes, supporting their role as strong alternatives when endoscopic management is not feasible. Open necrosectomy appears to carry the greatest burden of mortality, morbidity, and long‐term sequelae and should likely be reserved for carefully selected rescue settings.

Future multi‐center trials should also apply standardized and internationally accepted definitions for necrotizing pancreatitis phenotypes (e.g., infected vs. sterile necrosis and necrosis composition) and for procedural categories/step‐up algorithms. Only with such methodological standardization can truly robust and generalizable conclusions be drawn regarding the optimal interventional strategy.

## Author Contributions

**Ahmed Abdelsamad:** conceptualization, investigation, writing – original draft, methodology, validation, visualization, writing – review and editing, software, formal analysis, project administration, data curation, supervision, resources. **Jawad Alqedra:** methodology, investigation, writing – original draft, software, writing – review and editing, validation. **Ibrahim Khalil:** investigation, writing – original draft, methodology, formal analysis, software, data curation. **Mohammed Khaled Mohammed:** methodology, validation, visualization. **Osama Elsayed Mohammed Selim:** investigation, methodology, validation, visualization. **Amr Elserafy:** writing – original draft, writing – review and editing, supervision, data curation, visualization. **Omar A. Ahmed:** investigation, methodology, validation. **Mennatullah Mohsen:** methodology, validation, investigation. **Ahmed Elsherif:** methodology, validation, visualization, investigation. **Florian Gebauer:** supervision, writing – review and editing, visualization, validation, project administration. **Khaled Ashraf Mohamed:** supervision, resources, formal analysis, software, validation, visualization, writing – review and editing.

## Funding

The authors have nothing to report.

## Conflicts of Interest

The authors declare no conflicts of interest.

## Supporting information


Supporting Information S1

Supporting Information S2

## Data Availability

The data that support the findings of this study are available from the corresponding author upon reasonable request.

## References

[1] M. Werge , S. Novovic , P. N. Schmidt , and L. L. Gluud , “Infection Increases Mortality in Necrotizing Pancreatitis: A Systematic Review and Meta‐Analysis,” Pancreatology 16, no. 5 (2016): 698–707, 10.1016/j.pan.2016.07.004. 27449605

[2] M. Arvanitakis , J.‐M. Dumonceau , J. Albert , et al., “Endoscopic Management of Acute Necrotizing Pancreatitis: European Society of Gastrointestinal Endoscopy (ESGE) Evidence‐Based Multidisciplinary Guidelines,” Endoscopy 50, no. 5 (2018): 524–546, 10.1055/a-0588-5365. 29631305

[3] N. J. Schepers , O. J. Bakker , M. G. Besselink , et al., “Impact of Characteristics of Organ Failure and Infected Necrosis on Mortality in Necrotising Pancreatitis,” Gut 68, no. 6 (2019): 1044–1051, 10.1136/gutjnl-2017-314657. 29950344

[4] D. Paramythiotis , E. Karlafti , D. Tsavdaris , et al., “When to Intervene in Acute Necrotizing Pancreatitis: A Narrative Review of the Optimal Timing for Intervention Strategies,” Médica Sur 60, no. 10 (2024): 1592, 10.3390/medicina60101592. PMC11509130 39459378

[5] C. Armbruster and S. Kriwanek , “Early Versus Late Necrosectomy in Severe Necrotizing Pancreatitis,” American Journal of Surgery 175, no. 4 (1998): 341, PMID: 9568668, 10.1016/S0002-9610(96)00425-4. 9568668

[6] V. Arteaga Peralta , “Walled‐Off Pancreatic Necrosis,” in Acute Pancreatitis: Health Effects, Clinical Aspects and Emerging Therapies (Nova Science Publishers Inc., 2016), 33–54.

[7] T. K. Maatman and N. J. Z. Maatman , “Management of Necrotizing Pancreatitis,” Advances in Surgery 56, no. 1 (2022): 13–35, 10.1016/j.yasu.2022.02.010. 36096565

[8] L. R. Maurer and P. J. Fagenholz , “Contemporary Surgical Management of Pancreatic Necrosis,” JAMA Surgery 158, no. 1 (2023): 81–88, 10.1001/jamasurg.2022.5695. 36383374

[9] C. M. Luckhurst , M. El Hechi , A. E. Elsharkawy , et al., “Improved Mortality in Necrotizing Pancreatitis With a Multidisciplinary Minimally Invasive Step‐Up Approach: Comparison With a Modern Open Necrosectomy Cohort,” Journal of the American College of Surgeons 230, no. 6 (2020): 873–883, 10.1016/j.jamcollsurg.2020.01.038. 32251846

[10] R. Gupta , A. Kulkarni , R. Babu , et al., “Complications of Percutaneous Drainage in Step‐Up Approach for Management of Pancreatic Necrosis: Experience of 10 Years From a Tertiary Care Center,” Journal of Gastrointestinal Surgery 24, no. 3 (2020): 598–609, 10.1007/s11605-019-04470-z. 31845144

[11] R. P. Voermans , M. G. Besselink , and P. Fockens , “Endoscopic Management of Walled‐Off Pancreatic Necrosis,” Journal of Hepato‐Biliary‐Pancreatic Sciences 22, no. 1 (2015): 20–26, 10.1002/jhbp.180. 25345777

[12] A. Y. Li , J. R. Bergquist , and B. C. Visser , “Necrosectomy in the Management of Necrotizing Pancreatitis,” Advances in Surgery 55 (2021): 231–250, 10.1016/j.yasu.2021.05.016. 34389094

[13] J. A. Logue and C. R. Carter , “Minimally Invasive Necrosectomy Techniques in Severe Acute Pancreatitis: Role of Percutaneous Necrosectomy and Video‐Assisted Retroperitoneal Debridement,” Gastroenterology Research and Practice 2015 (2015): 1–6, 10.1155/2015/693040. PMC4637484 26587018

[14] R. A. Hollemans , S. van Brunschot , O. J. Bakker , et al., “Minimally Invasive Intervention for Infected Necrosis in Acute Pancreatitis,” Expert Review of Medical Devices 11, no. 6 (2014): 637–648, 10.1586/17434440.2014.947271. 25122506

[15] H. L. Husu , J. A. Kuronen , A. K. Leppäniemi , and P. J. Mentula , “Open Necrosectomy in Acute Pancreatitis‐Obsolete or Still Useful?,” World Journal of Emergency Surgery 15, no. 1 (2020): 21, 10.1186/s13017-020-00300-9. 32183878 PMC7079510

[16] J. Y. Bang , S. Lakhtakia , S. Thakkar , et al., “Upfront Endoscopic Necrosectomy or Step‐Up Endoscopic Approach for Infected Necrotising Pancreatitis (DESTIN): A Single‐Blinded, Multicentre, Randomised Trial,” Lancet Gastroenterology & Hepatology 9, no. 1 (2024): 22–33, 10.1016/S2468-1253(23)00331-X. 37980922

[17] A. Rosenberg , E. A. Steensma , and L. M. Napolitano , “Necrotizing Pancreatitis: New Definitions and a New Era in Surgical Management,” Surgical Infections 16, no. 1 (2015): 1–13, 10.1089/sur.2014.123. 25761075

[18] M. C. Van Baal , H. C. Van Santvoort , T. L. Bollen , O. J. Bakker , M. G. Besselink , and H. G. Gooszen , “Systematic Review of Percutaneous Catheter Drainage as Primary Treatment for Necrotizing Pancreatitis,” British Journal of Surgery 98, no. 1 (2011): 18–27, 10.1002/bjs.7304. 21136562

[19] J. Werner , W. Hartwig , T. Hackert , and M. W. Büchler , “Surgery in the Treatment of Acute Pancreatitis—Open Pancreatic Necrosectomy,” Scandinavian Journal of Surgery 94, no. 2 (2005): 130–134, 10.1177/145749690509400209. 16111095

[20] T. B. Gardner , P. Chahal , G. I. Papachristou , et al., “A Comparison of Direct Endoscopic Necrosectomy With Transmural Endoscopic Drainage for the Treatment of Walled‐Off Pancreatic Necrosis,” Gastrointestinal Endoscopy 69, no. 6 (2009): 1085–1094, 10.1016/j.gie.2008.06.061. 19243764

[21] B. Hutton , G. Salanti , D. M. Caldwell , et al., “The PRISMA Extension Statement for Reporting of Systematic Reviews Incorporating Network Meta‐Analyses of Health Care Interventions: Checklist and Explanations,” Annals of Internal Medicine 162, no. 11 (2015): 777–784, 10.7326/M14-2385. 26030634

[22] J. P. T. Higgins , J. Thomas , J. Chandler , et al., “Cochrane Handbook for Systematic Reviews of Interventions,” in Cochrane Handbook for Systematic Reviews of Interventions (2019): 1–694, 10.1002/9781119536604. PMC10284251 31643080

[23] C. Schardt , M. B. Adams , T. Owens , S. Keitz , and P. Fontelo , “Utilization of the PICO Framework to Improve Searching PubMed for Clinical Questions,” BMC Medical Informatics and Decision Making 7, no. 1 (December 2007): 16, 10.1186/1472-6947-7-16. 17573961 PMC1904193

[24] I. W. Group , “IAP/APA Evidence‐Based Guidelines for the Management of Acute Pancreatitis,” supplement, Pancreatology 13, no. S4 (2013): e1–e15, 10.1016/j.pan.2013.07.063. 24054878

[25] B. P. T. Loveday , J. I. Rossaak , A. Mittal , A. Phillips , and J. A. Windsor , “Survey of Trends in Minimally Invasive Intervention for Necrotizing Pancreatitis,” ANZ Journal of Surgery 81, no. 1–2 (January 2011): 56–64, 10.1111/j.1445-2197.2010.05265.x. 21299800

[26] G. Rücker , “Network Meta‐Analysis, Electrical Networks and Graph Theory,” Research Synthesis Methods 3, no. 4 (2012): 312–324, 10.1002/jrsm.1058. 26053424

[27] J. Arredondo Montero , “Interpreting Heterogeneity in Meta‐Analysis: A Unified Framework Across Intervention, Diagnostic, and Prognostic Reviews,” Preprints (September 2025), 10.20944/preprints202508.1527.v2.

[28] D. Bausch , U. Wellner , S. Kahl , et al., “Minimally Invasive Operations for Acute Necrotizing Pancreatitis: Comparison of Minimally Invasive Retroperitoneal Necrosectomy With Endoscopic Transgastric Necrosectomy,” Surgery 152, no. 3 (September 2012): S128–S134, 10.1016/j.surg.2012.05.021. 22770962

[29] G. Rücker and G. Schwarzer , “Ranking Treatments in Frequentist Network Meta‐Analysis Works Without Resampling Methods,” BMC Medical Research Methodology 15, no. 1 (2015): 58, 10.1186/s12874-015-0060-8. 26227148 PMC4521472

[30] S. S. Qian , Environmental and Ecological Statistics with R (Chapman and Hall/CRC, 2009), 10.1201/b17172.

[31] R. Grant and G. L. Di Tanna , Bayesian Meta‐Analysis (Chapman and Hall/CRC, 2025), 10.1201/9781003375821.

[32] H. Wickham , “ggplot2,” WIREs Computational Statistics 3, no. 2 (March 2011): 180–185, 10.1002/wics.147.

[33] “Google LLC,” NotebookLM, accessed March, 2026, https://notebooklm.google/.

[34] H. C. van Santvoort , M. G. Besselink , O. J. Bakker , et al., “A Step‐Up Approach or Open Necrosectomy for Necrotizing Pancreatitis,” New England Journal of Medicine 362, no. 16 (April 2010): 1491–1502, 10.1056/NEJMoa0908821. 20410514

[35] O. J. Bakker , H. C. van Santvoort , S. van Brunschot , et al., “Endoscopic Transgastric vs Surgical Necrosectomy for Infected Necrotizing Pancreatitis,” JAMA 307, no. 10 (March 2012): 1053, 10.1001/jama.2012.276. 22416101

[36] S. van Brunschot , J. van Grinsven , H. C. van Santvoort , et al., “Endoscopic or Surgical Step‐Up Approach for Infected Necrotising Pancreatitis: A Multicentre Randomised Trial,” Lancet 391, no. 10115 (January 2018): 51–58, 10.1016/S0140-6736(17)32404-2. 29108721

[37] J. Y. Bang , J. P. Arnoletti , B. A. Holt , et al., “An Endoscopic Transluminal Approach, Compared With Minimally Invasive Surgery, Reduces Complications and Costs for Patients With Necrotizing Pancreatitis,” Gastroenterology 156, no. 4 (March 2019): 1027–1040.e3, 10.1053/j.gastro.2018.11.031. 30452918

[38] M. Avudiappan , V. Bhargava , A. Kulkarni , M. Kang , S. S. Rana , and R. Gupta , “Evaluating the Role of the Minimal Incision Retroperitoneal Necrosectomy (MIRN) in the Management of Infected Pancreatic Necrosis: Experience From a Tertiary Care Center,” Surgery Open Science 15 (2023): 38–42, 10.1016/j.sopen.2023.07.004. 37609368 PMC10440548

[39] N. Kumar , D. L. Conwell , and C. C. Thompson , “Direct Endoscopic Necrosectomy Versus Step‐Up Approach for Walled‐Off Pancreatic Necrosis,” Pancreas 43, no. 8 (November 2014): 1334–1339, 10.1097/MPA.0000000000000213. 25083997 PMC5019103

[40] Y. Tu , H. Jiao , X. Tan , L. Sun , and W. Zhang , “Laparotomy Versus Retroperitoneal Laparoscopy in Debridement and Drainage of Retroperitoneal Infected Necrosis in Severe Acute Pancreatitis,” Surgical Endoscopy 27, no. 11 (November 2013): 4217–4223, 10.1007/s00464-013-3026-0. 23793802

[41] H. C. Van Santvoort , M. G. H. Besselink , K. D. Horvath , et al., “Videoscopic Assisted Retroperitoneal Debridement in Infected Necrotizing Pancreatitis,” HPB 9, no. 2 (April 2007): 156–159, 10.1080/13651820701225688. 18333133 PMC2020795

[42] L. Yan , A Dargan , J Nieto , et al., “Direct Endoscopic Necrosectomy at the Time of Transmural Stent Placement Results in Earlier Resolution of Complex Walled‐Off Pancreatic Necrosis: Results From a Large Multicenter United States Trial,” Endoscopic Ultrasound 8, no. 3 (2019): 172, 10.4103/eus.eus_108_17. 29882517 PMC6590004

[43] P. K. Garg , D. Meena , D. Babu , et al., “Endoscopic Versus Laparoscopic Drainage of Pseudocyst and Walled‐Off Necrosis Following Acute Pancreatitis: A Randomized Trial,” Surgical Endoscopy 34, no. 3 (March 2020): 1157–1166, 10.1007/s00464-019-06866-z. 31140002

[44] N. A. Kumta , A. Tyberg , V. H. Bhagat , et al., “EUS‐Guided Drainage of Pancreatic Fluid Collections by Lumen Apposing Metal Stents: An International, Multicenter Experience,” Digestive and Liver Disease 51, no. 11 (2019): 1557–1561, 10.1016/j.dld.2019.05.029. 31272934

[45] N. Oblizajek , N. Takahashi , S. Agayeva , et al., “Outcomes of Early Endoscopic Intervention for Pancreatic Necrotic Collections: A Matched Case‐Control Study,” Gastrointestinal Endoscopy 91, no. 6 (2020): 1303–1309, 10.1016/j.gie.2020.01.036. 31958461

[46] J. Guo , A. Saftoiu , P. Vilmann , et al., “A Multi‐Institutional Consensus on How to Perform Endoscopic Ultrasound‐Guided Peri‐Pancreatic Fluid Collection Drainage and Endoscopic Necrosectomy,” Endoscopic Ultrasound 6, no. 5 (2017): 285–291, 10.4103/eus.eus_85_17. 29063871 PMC5664848

[47] S. Jain and A. Hasan , “Predictors of Step up Approach Using EUS‐Guided Transmural Drainage for Walled‐Off Necrosis,” Gastrointestinal Endoscopy (2019), 10.1016/j.gie.2019.03.039.

[48] M. A. Al Efishat , M. A. Schattner , H. Gerdes , et al., “Endoscopic Versus Percutaneous Drainage of Post‐Operative Peripancreatic Fluid Collections Following Pancreatic Resection,” HPB 21, no. 4 (2019): 434–443, 10.1016/j.hpb.2018.08.010. 30293867 PMC7570452

[49] M. Takenaka , K. Minaga , K. Kamata , et al., “Clinical Outcomes of EUS‐Guided Drainage for Pancreatic Walled‐Off Necrosis,” Digestive and Liver Disease 53, no. 4 (2021): 499–505, 10.1016/j.dld.2020.10.013.

[50] R. Dhingra , S. Srivastava , A. Behari , et al., “Percutaneous Endoscopic Necrosectomy Under Conscious Sedation,” Journal of Gastroenterology and Hepatology 30, no. 8 (2015): 1271–1276, 10.1111/jgh.12821.

[51] M. Saumoy , N. A. Kumta , A. Engel , et al., “Endoscopic Treatment of Walled‐Off Pancreatic Necrosis,” Surgical Endoscopy 32, no. 5 (2018): 2484–2491, 10.1007/s00464-017-5960-4.

[52] S. Mangiafico , G. Lauri , F. Prato , et al., “EUS‐Guided Drainage Using LAMS for Pancreatic Walled‐Off Necrosis,” Digestive and Liver Disease 56, no. 2 (2024): 267–273, 10.1016/j.dld.2023.09.012.

[53] E. Tenorio González , N. El‐Domiaty , E. Ragot , et al., “Hybrid Percutaneous‐Endoscopic Necrosectomy for Large Walled‐Off Pancreatic Necrosis ‐ An Observational Feasibility Study,” Revista Española de Enfermedades Digestivas 118, no. 4 (2026): 204–210, 10.17235/reed.2025.11510/2025. 41020962

[54] F. Liu , L. Wu , J. Guo , et al., “Video‐Assisted Retroperitoneal Debridement for Infected Pancreatic Necrosis,” Surgery 168, no. 2 (2020): 317–322, 10.1016/j.surg.2019.05.057.

[55] J. Wan , D. Wu , W. He , et al., “Step‐up Minimally Invasive Approach for Infected Necrotizing Pancreatitis,” Asian Journal of Surgery 47, no. 2 (2024): 924–930, 10.1016/j.asjsur.2023.11.088.

[56] A. Li , F. Cao , J. Li , et al., “Step‐Up Mini‐Invasive Surgery for Infected Pancreatic Necrosis,” Surgical Endoscopy 30, no. 5 (2016): 1846–1853, 10.1007/s00464-015-4507-0.

[57] Q. Wu , J. Hu , S. Peng , et al., “Minimally Invasive Step‐Up Approach Versus Open Necrosectomy,” Surgical Endoscopy 36, no. 5 (2022): 3420–3429, 10.1007/s00464-021-08828-y.

[58] D. Budkule , B. Pottakkat , R. Ballari , et al., “VARD in Infected Pancreatic Necrosis,” International Journal of Surgery 67 (2019): 29–33, 10.1016/j.ijsu.2019.05.001.

[59] C. Chaves , J. Mesquita , P. Torres , et al., “VARD for Pancreatic Necrosis: Outcomes and Predictors,” Cirugia Espanola 102, no. 1 (2024): 25–31, 10.1016/j.cireng.2023.10.012.38141845

[60] M. A. Garcia‐Urena , J. Lopez‐Monclus , D. Melero , et al., “Outcomes of Open Necrosectomy for Infected Pancreatic Necrosis,” Surgical Endoscopy 27, no. 4 (2013): 1264–1270, 10.1007/s00464-012-2592-3.

[61] R. M. Eickhoff , J. Steinbusch , P. Seppelt , et al., “Videoassistiertes Retroperitoneales Débridement,” Der Chirurg 88, no. 9 (2017): 785–791, 10.1007/s00104-017-0377-4. 28180976

[62] S. Ulagendra Perumal , A. Naveen , S. Perumal , J. Sathyanesan , and R. Palaniappan , “Outcome After Open Pancreatic Necrosectomy,” Journal of Minimal Access Surgery 9, no. 3 (2013): 124–129, 10.1111/ans.12107.

[63] A. L. Wei , Q. Guo , M. J. Wang , et al., “Early Versus Delayed Open Necrosectomy for Infected Necrotizing Pancreatitis,” Surgery 173, no. 4 (2023): 1011–1016, 10.1016/j.surg.2022.12.015.

[64] A. Sileikis , V. Beiša , A. Beiša , et al., “Minimally Invasive Retroperitoneal Necrosectomy in Acute Necrotizing Pancreatitis,” BMC Surgery 12 (2012): 11, 10.1186/1471-2482-12-13. 23630551 PMC3627149

[65] M. A. Ebrahim , H. Sato , T. Kato , et al., “Efficacy and Safety of Endoscopic Necrosectomy for Walled‐Off Necrosis,” Digestive Endoscopy 34, no. 6 (2022): 1205–1213, 10.1111/den.14199.35318750

[66] M. J. Page , J. E. McKenzie , P. M. Bossuyt , et al., “The PRISMA 2020 Statement: An Updated Guideline for Reporting Systematic Reviews,” BMJ 372 (2021): n71, 10.1136/bmj.n71. 33782057 PMC8005924

[67] J. Y. Bang , C. M. Wilcox , J. P. Arnoletti , and S. Varadarajulu , “Superiority of Endoscopic Interventions Over Minimally Invasive Surgery for Infected Necrotizing Pancreatitis: Meta‐Analysis of Randomized Trials,” Digestive Endoscopy 32, no. 3 (March 2020): 298–308, 10.1111/den.13470. 31220368

[68] A. M. Onnekink , L. Boxhoorn , H. C. Timmerhuis , et al., “Endoscopic Versus Surgical Step‐Up Approach for Infected Necrotizing Pancreatitis (ExTENSION): Long‐Term Follow‐Up of a Randomized Trial,” Gastroenterology 163, no. 3 (September 2022): 712–722, 10.1053/j.gastro.2022.05.015. 35580661

[69] G. D. Tebala , F. Massimi , F. Duro , et al., “Single‐Loop Versus Double‐Loop Reconstruction After Pancreatoduodenectomy: Does It Impact on the Risk of Postoperative Pancreatic Fistula?,” Annals of Hepato‐Biliary‐Pancreatic Surgery (March 2026), 10.14701/ahbps.26-007. PMC13212024 41844339

[70] C. M. Haney , K. F. Kowalewski , M. W. Schmidt , et al., “Endoscopic Versus Surgical Treatment for Infected Necrotizing Pancreatitis: A Systematic Review and Meta‐Aalysis of Randomized Controlled Trials,” Surgical Endoscopy 34, no. 6 (June 2020): 2429–2444, 10.1007/s00464-020-07469-9. 32112252 PMC7214487

[71] A. Bruni , L. Colecchia , G. Dell’Anna , et al., “Nutritional Management in Chronic Pancreatitis: From Exocrine Pancreatic Insufficiency to Precision Therapy,” Nutrients 17, no. 17 (August 2025): 2720, 10.3390/nu17172720. 40944111 PMC12430654

[72] T. H. Baron , C. J. DiMaio , A. Y. Wang , and K. A. Morgan , “American Gastroenterological Association Clinical Practice Update: Management of Pancreatic Necrosis,” Gastroenterology 158, no. 1 (January 2020): 67–75.e1, 10.1053/j.gastro.2019.07.064. 31479658

[73] A. Abdelsamad , M. K. Mohammed , I. Khalil , et al., “Continuous vs. Interrupted Suturing in Hepaticojejunostomy: A Comprehensive Systematic Review and Meta‐Analysis,” Langenbeck’s Archives of Surgery 410, no. 1 (July 2025): 214, 10.1007/s00423-025-03756-y. PMC12227507 40613910

[74] I. Dumitrascu , N. O. Zarnescu , E. C. Zarnescu , et al., “Acute Necrotizing Pancreatitis—Advances and Challenges in Management for Optimal Clinical Outcomes,” Medicina (B. Aires) 61, no. 7 (June 2025): 1186, 10.3390/medicina61071186. PMC12300309 40731816

[75] T. K. Maatman , S. Mahajan , A. M. Roch , et al., “High Rates of Readmission in Necrotizing Pancreatitis: Natural History or Opportunity for Improvement?,” Journal of Gastrointestinal Surgery 23, no. 9 (September 2019): 1834–1839, 10.1007/s11605-018-04097-6. 30706374

[76] A. Abdelsamad , E. Ibrahim , A. Elsheikh , et al., “Worse Cholecystectomy Outcomes During the COVID‐19 Pandemic: Were Staff Shortages or a Change in Patient Case‐Mix the Culprit?,” Surgical Endoscopy 38, no. 12 (December 2024): 7389–7398, 10.1007/s00464-024-11337-1. 39443378

