# Supplementary material for: Comparative Outcomes of Endoscopic, Minimally Invasive Surgical, and Open Necrosectomy in Necrotizing Pancreatitis: Evidence From a Network Meta‐Analysis
Source: World J Surg. 2026 Jun 2;50(7):1875–90. doi: 10.1002/wjs.70406 (PMC13356536; doi:10.1002/wjs.70406)
Supplement: Supplementary file 2 — Supporting Information S1 [file WJS-50-1875-s002.docx]

**SUPPLEMENTARY FIGURES**

## Figure S1. Network Geometry Plots

Node size = total sample size; edge thickness = number of direct comparisons.

## Figure S1A. Network Geometry: Mortality


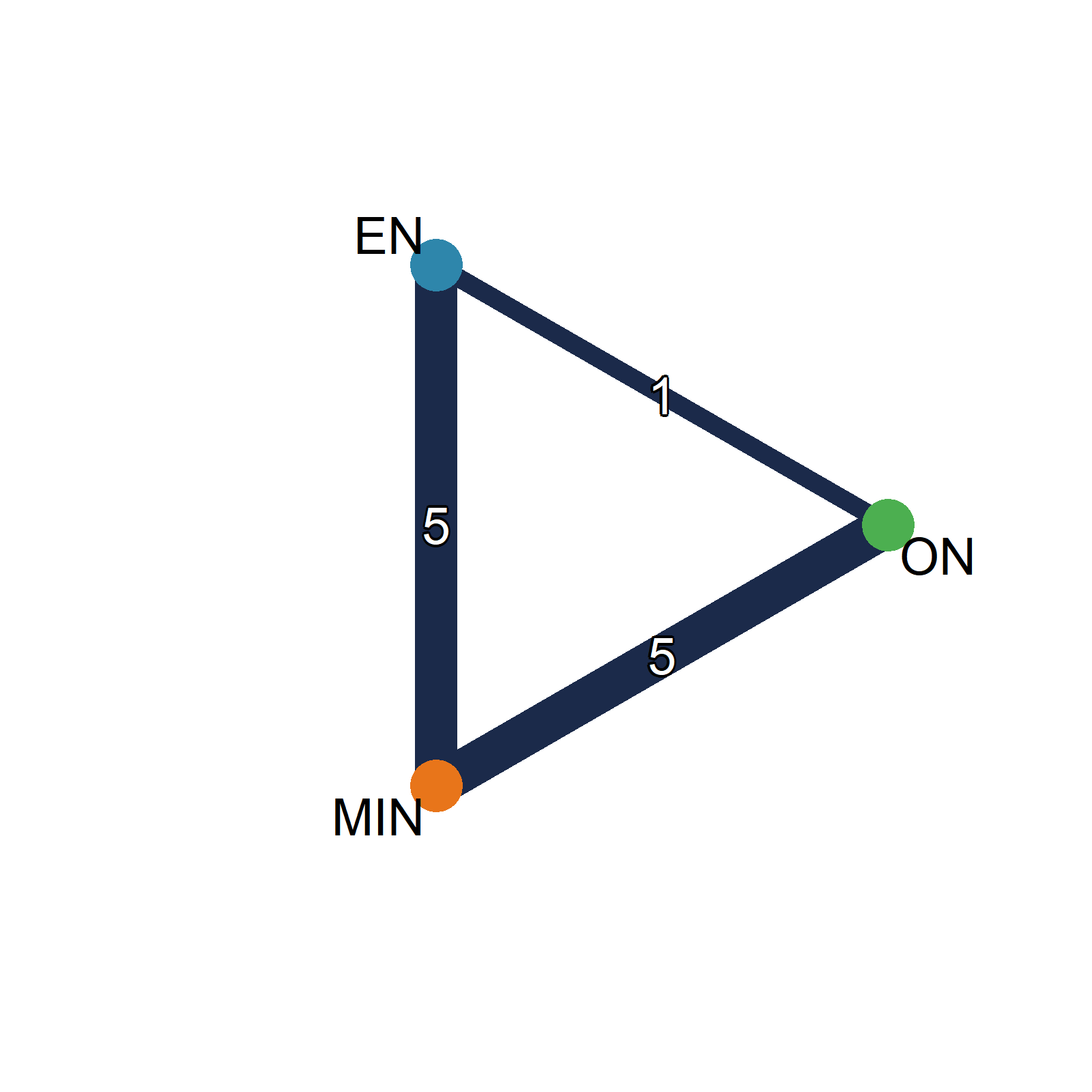


*Figure S1A. Network geometry for mortality. Three nodes: EN (endoscopic necrosectomy), MIN (minimally invasive necrosectomy), ON (open necrosectomy).*

## Figure S1B. Network Geometry: Complications


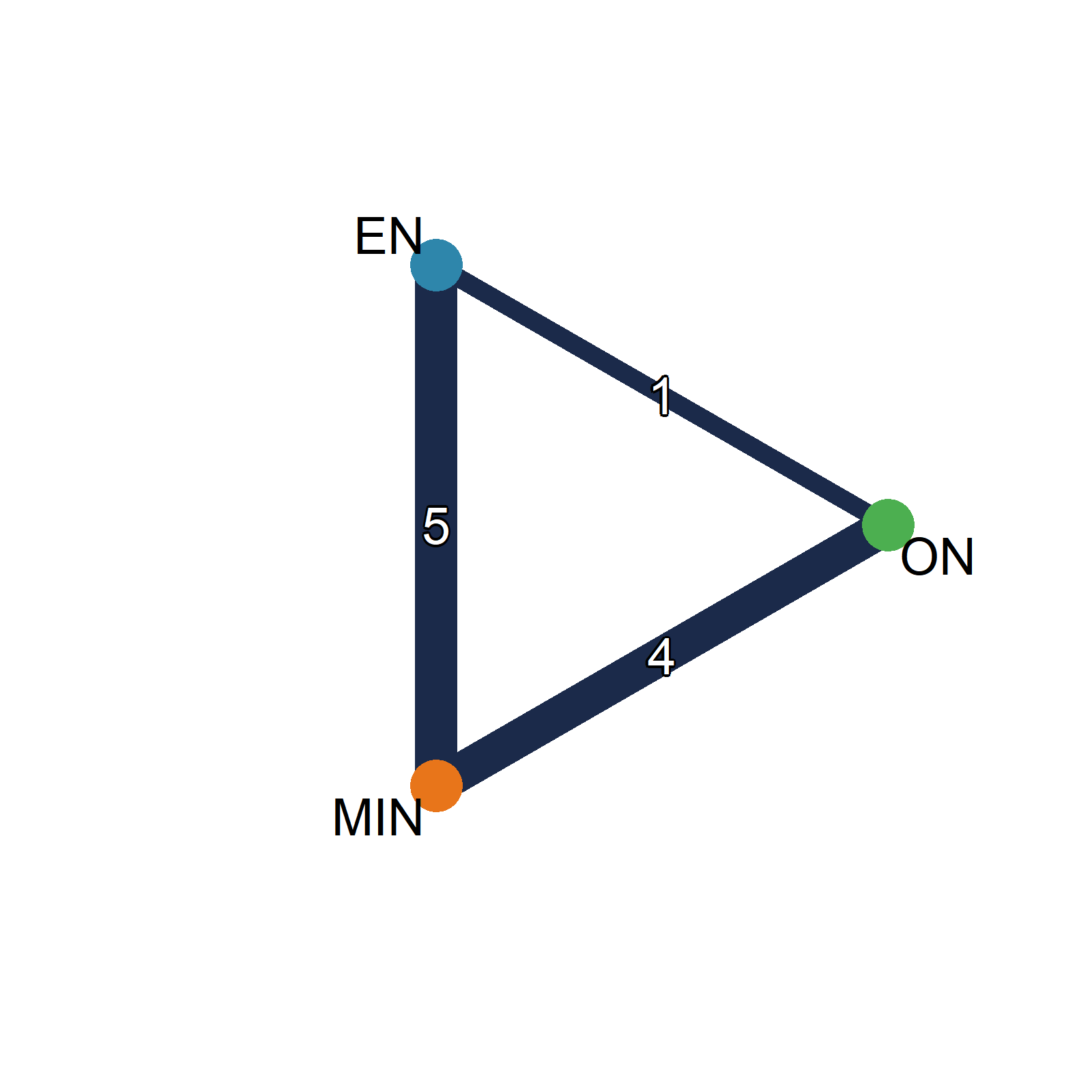


*Figure S1B. Network geometry for complications. Three nodes: EN (endoscopic necrosectomy), MIN (minimally invasive necrosectomy), ON (open necrosectomy).*

## Figure S1C. Network Geometry: New-Onset MOF


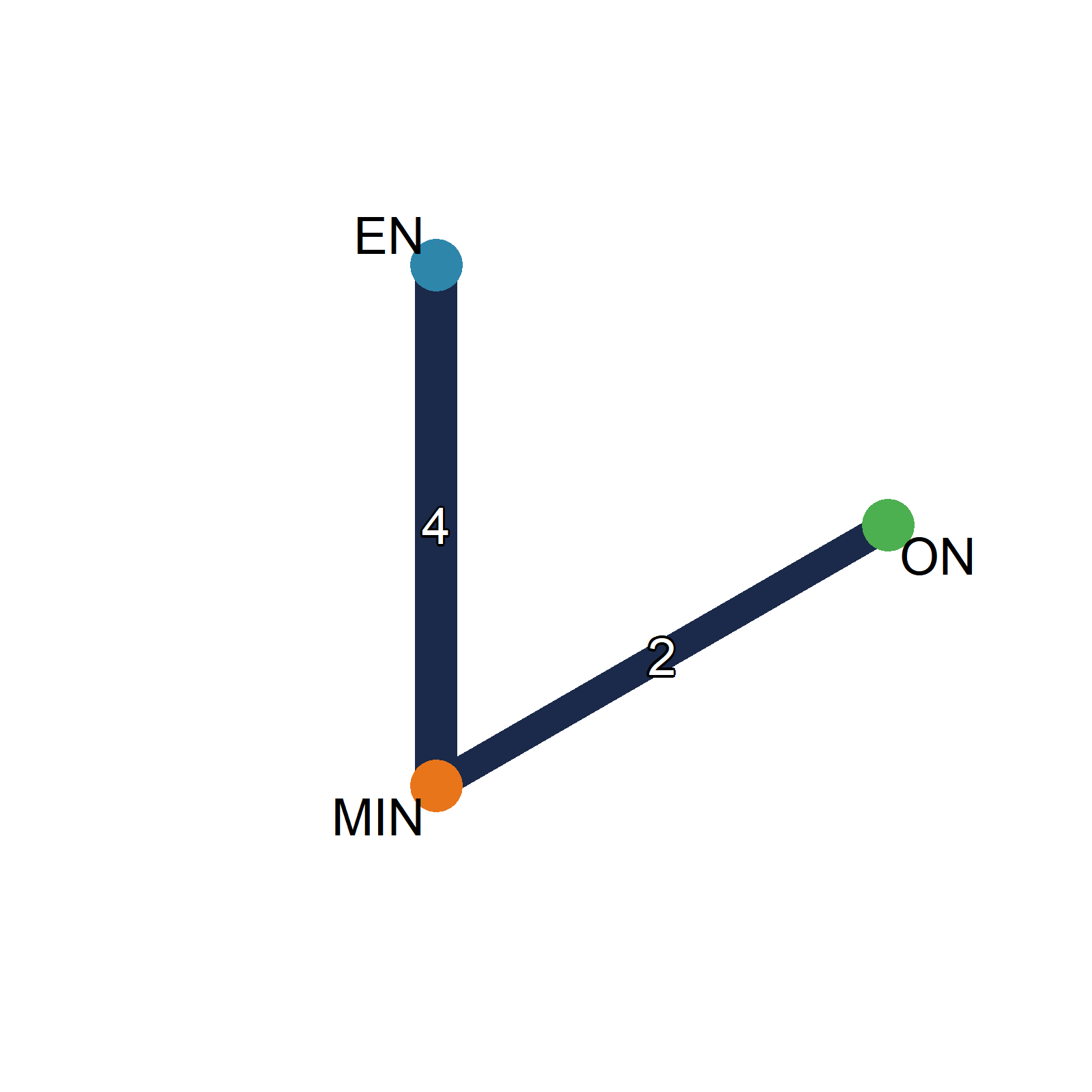


*Figure S1C. Network geometry for new-onset mof. Three nodes: EN (endoscopic necrosectomy), MIN (minimally invasive necrosectomy), ON (open necrosectomy).*

## Figure S1D. Network Geometry: Bleeding


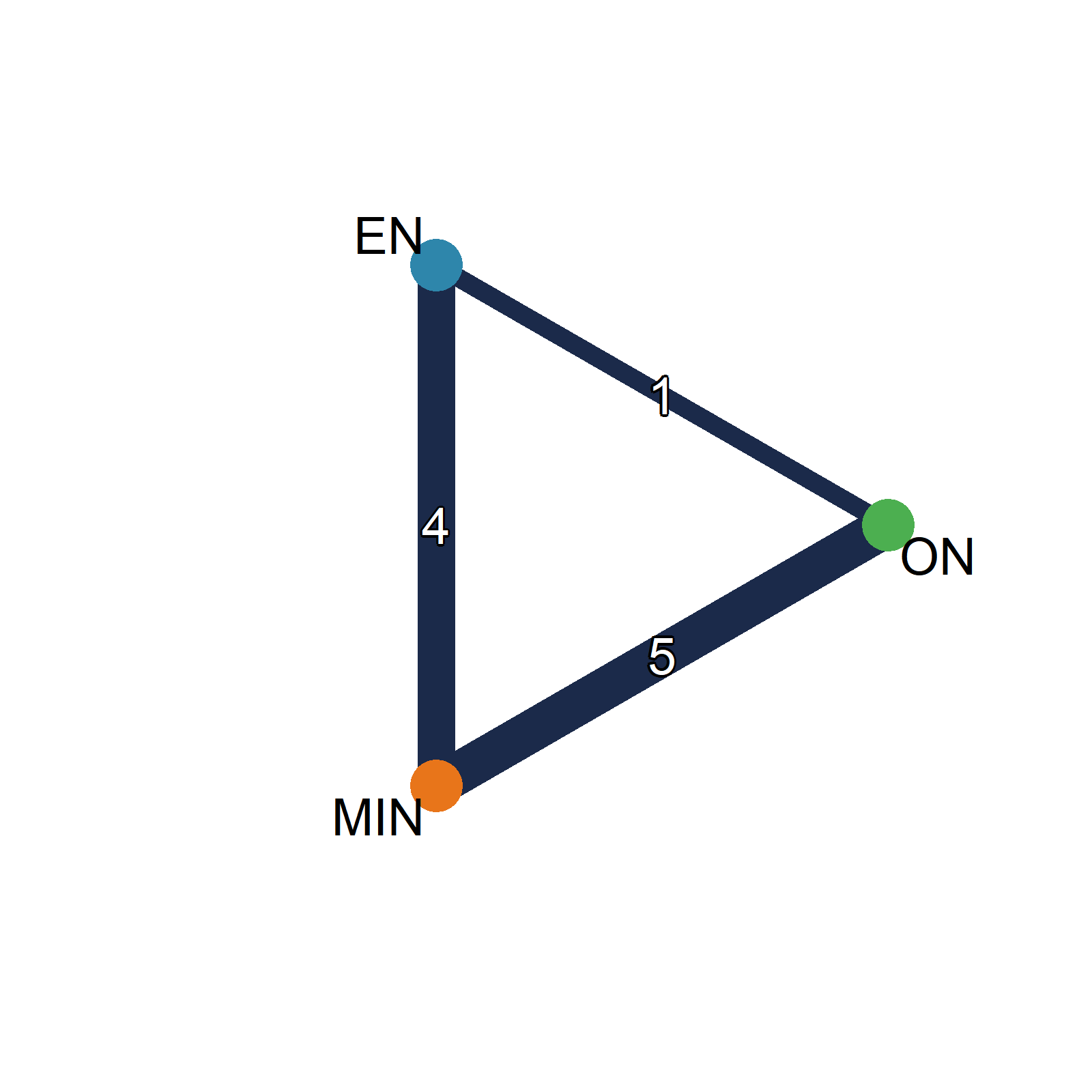


*Figure S1D. Network geometry for bleeding. Three nodes: EN (endoscopic necrosectomy), MIN (minimally invasive necrosectomy), ON (open necrosectomy).*

## Figure S1E. Network Geometry: New-Onset Diabetes


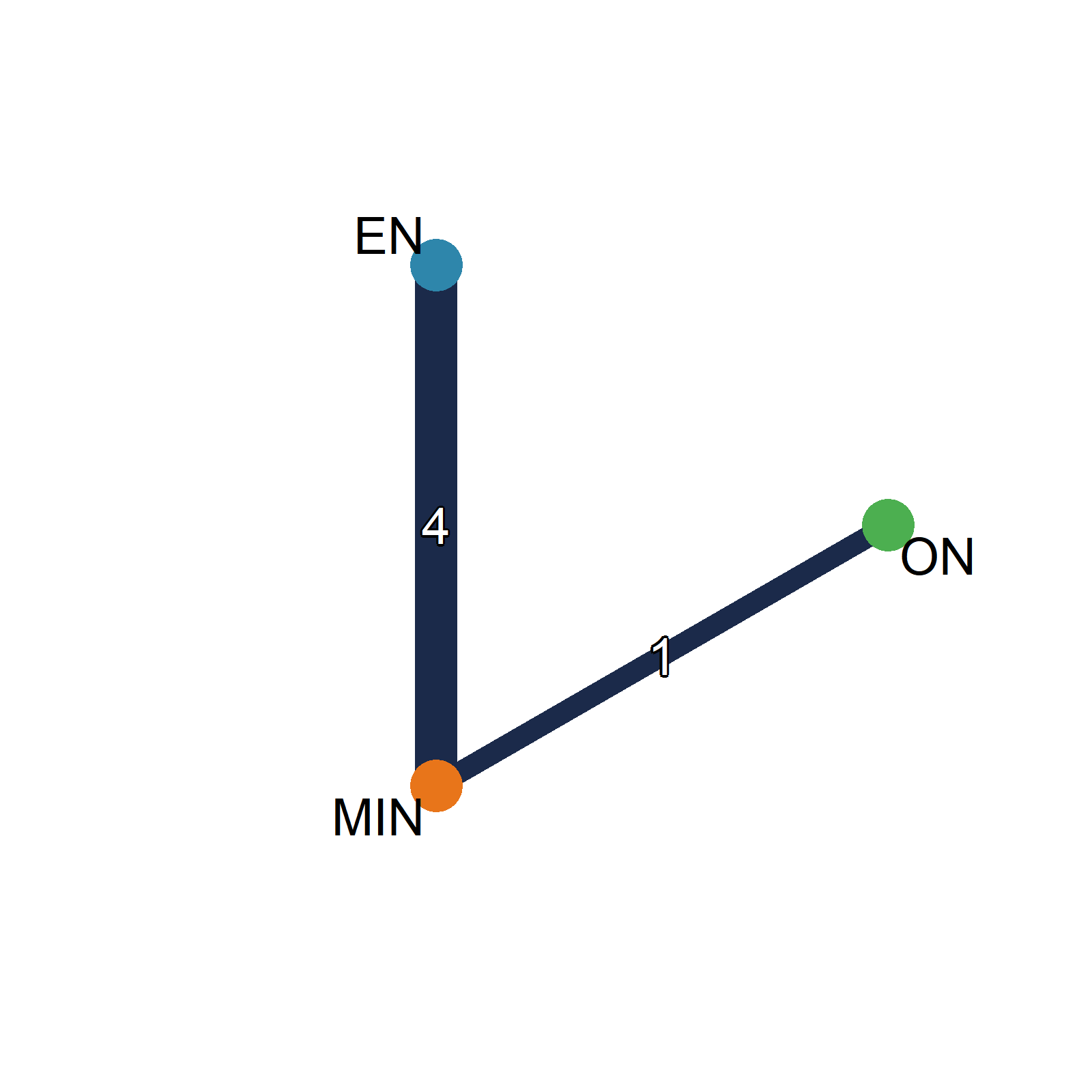


*Figure S1E. Network geometry for new-onset diabetes. Three nodes: EN (endoscopic necrosectomy), MIN (minimally invasive necrosectomy), ON (open necrosectomy).*

## Figure S1F. Network Geometry: Exocrine Insufficiency


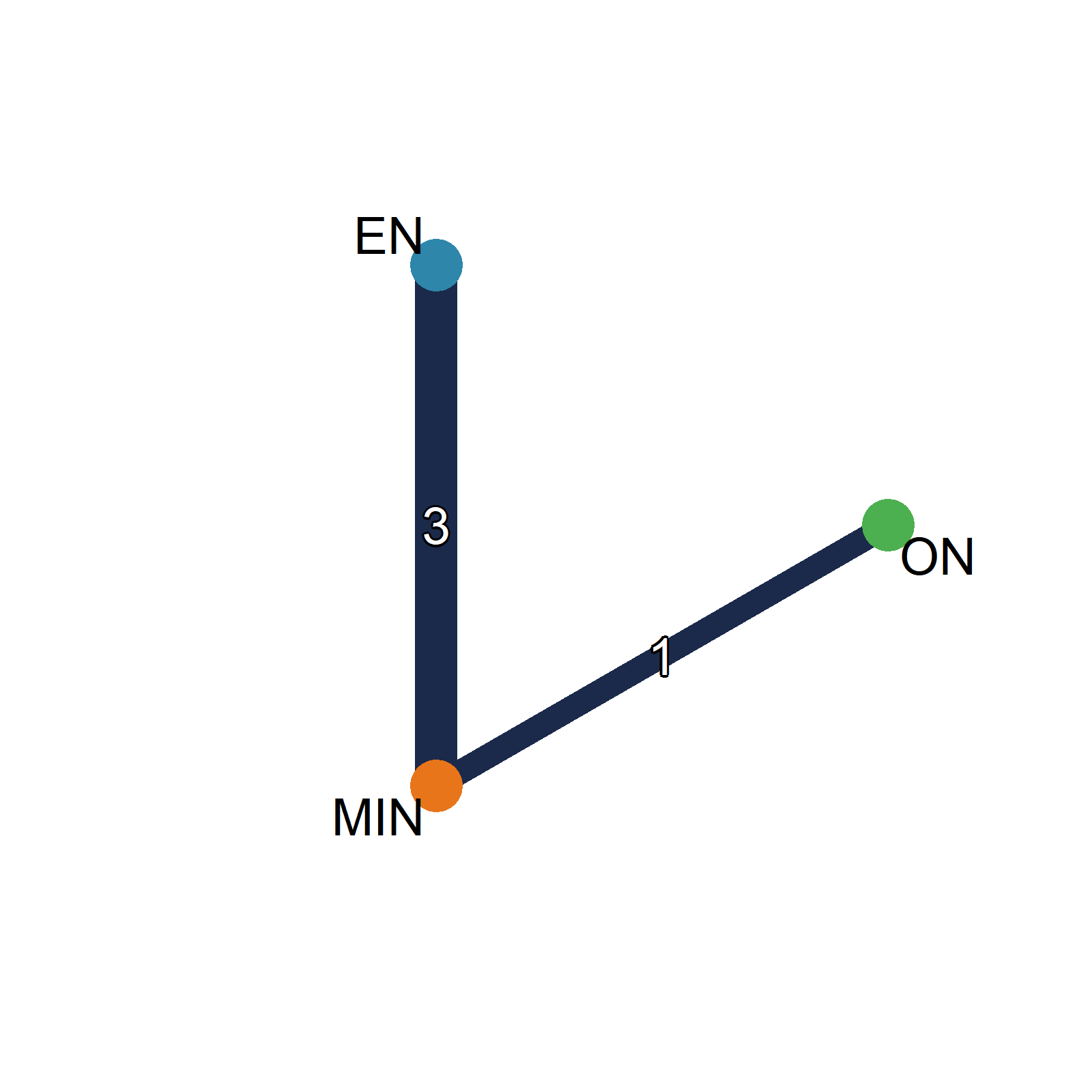


*Figure S1F. Network geometry for exocrine insufficiency. Three nodes: EN (endoscopic necrosectomy), MIN (minimally invasive necrosectomy), ON (open necrosectomy).*

## Figure S1G. Network Geometry: Reintervention


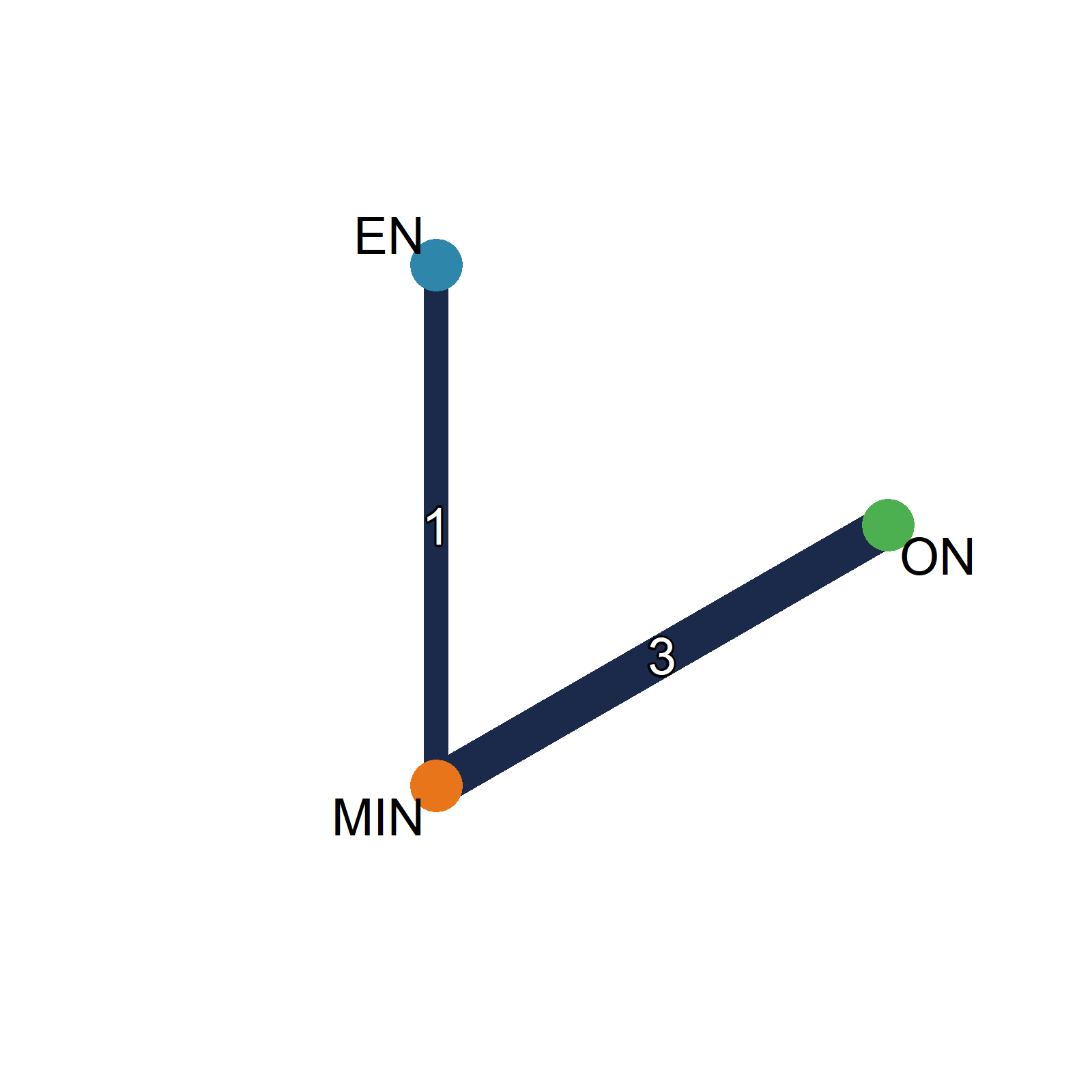


*Figure S1G. Network geometry for reintervention. Three nodes: EN (endoscopic necrosectomy), MIN (minimally invasive necrosectomy), ON (open necrosectomy).*

## Figure S1H. Network Geometry: Incisional Hernia


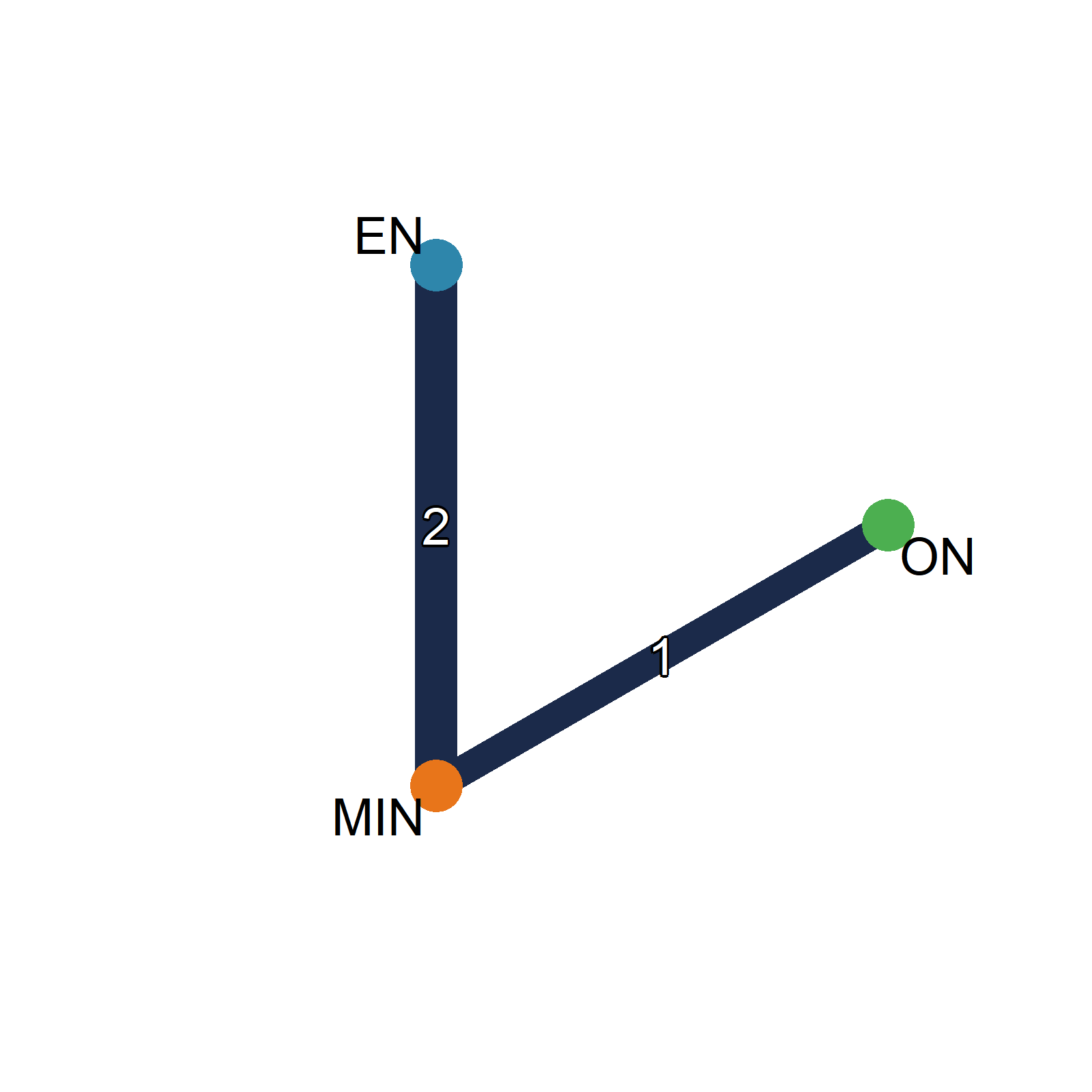


*Figure S1H. Network geometry for incisional hernia. Three nodes: EN (endoscopic necrosectomy), MIN (minimally invasive necrosectomy), ON (open necrosectomy).*

## Figure S1I. Network Geometry: New ICU Admission


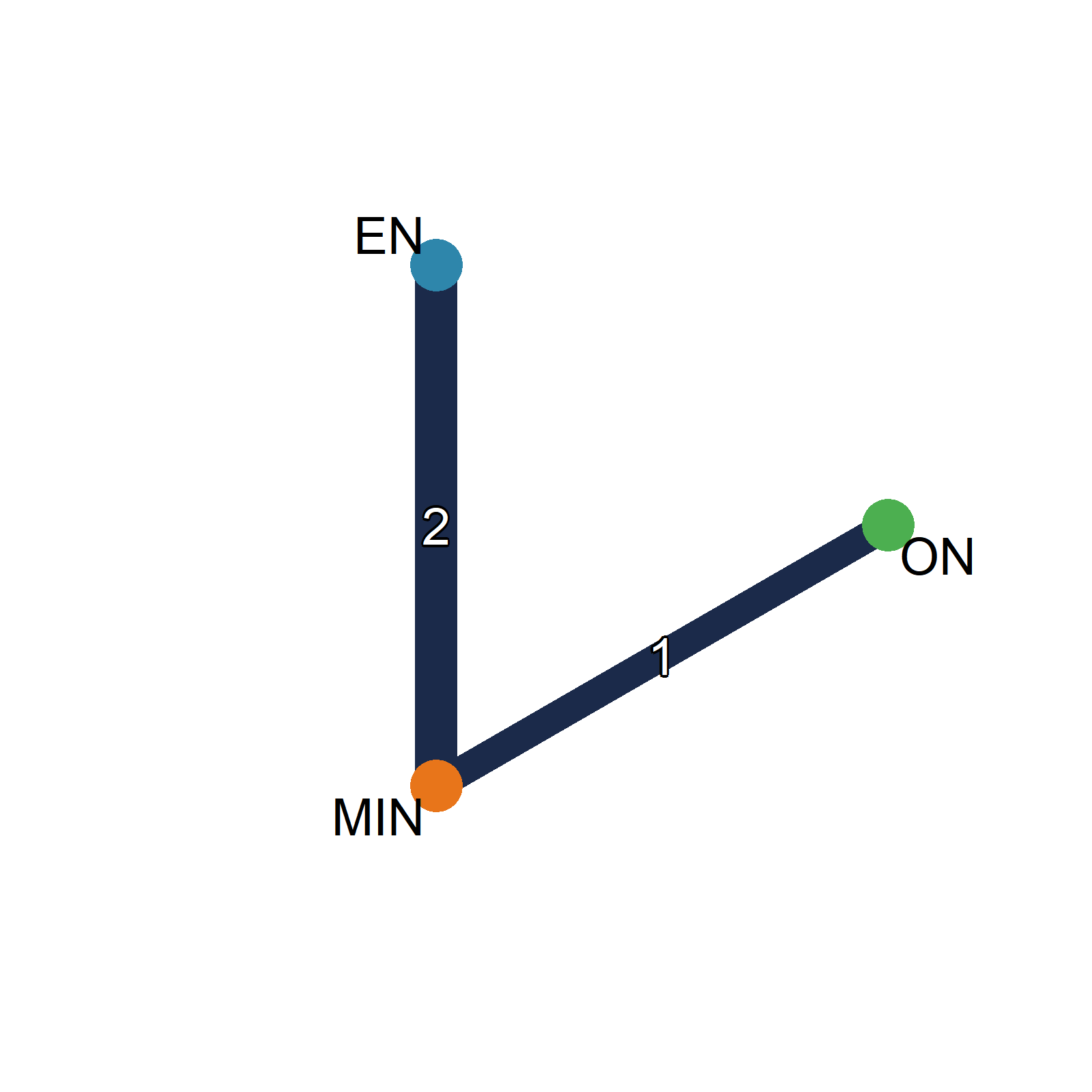


*Figure S1I. Network geometry for new icu admission. Three nodes: EN (endoscopic necrosectomy), MIN (minimally invasive necrosectomy), ON (open necrosectomy).*

## Figure S2. Node-Splitting (Consistency) Analysis

Comparison of direct and indirect evidence for each treatment pair.

## Figure S2A. Node-Split: Mortality


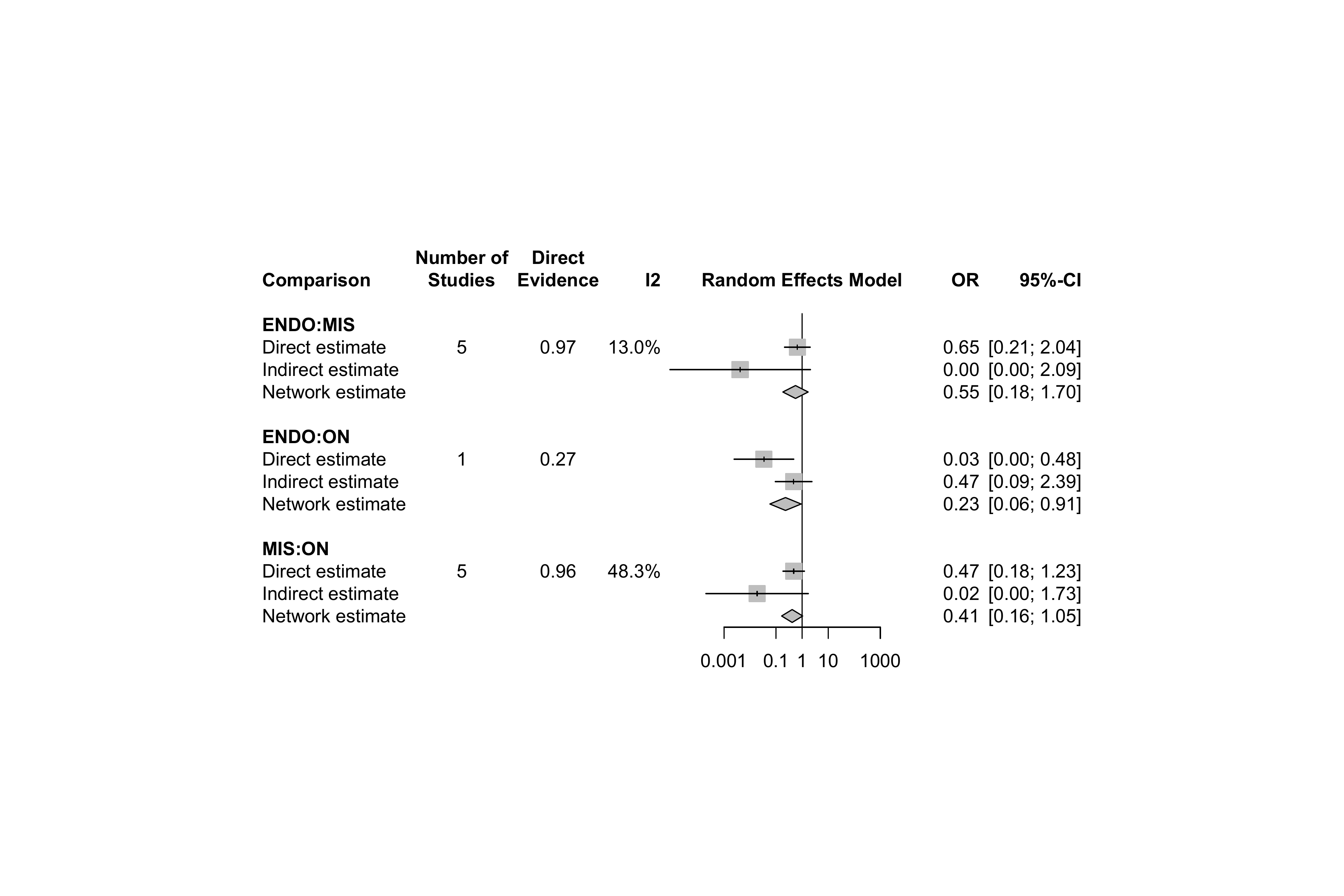


*Figure S2A. Node-splitting analysis for mortality. Direct and indirect estimates compared. No significant inconsistency detected.*

## Figure S2B. Node-Split: Complications


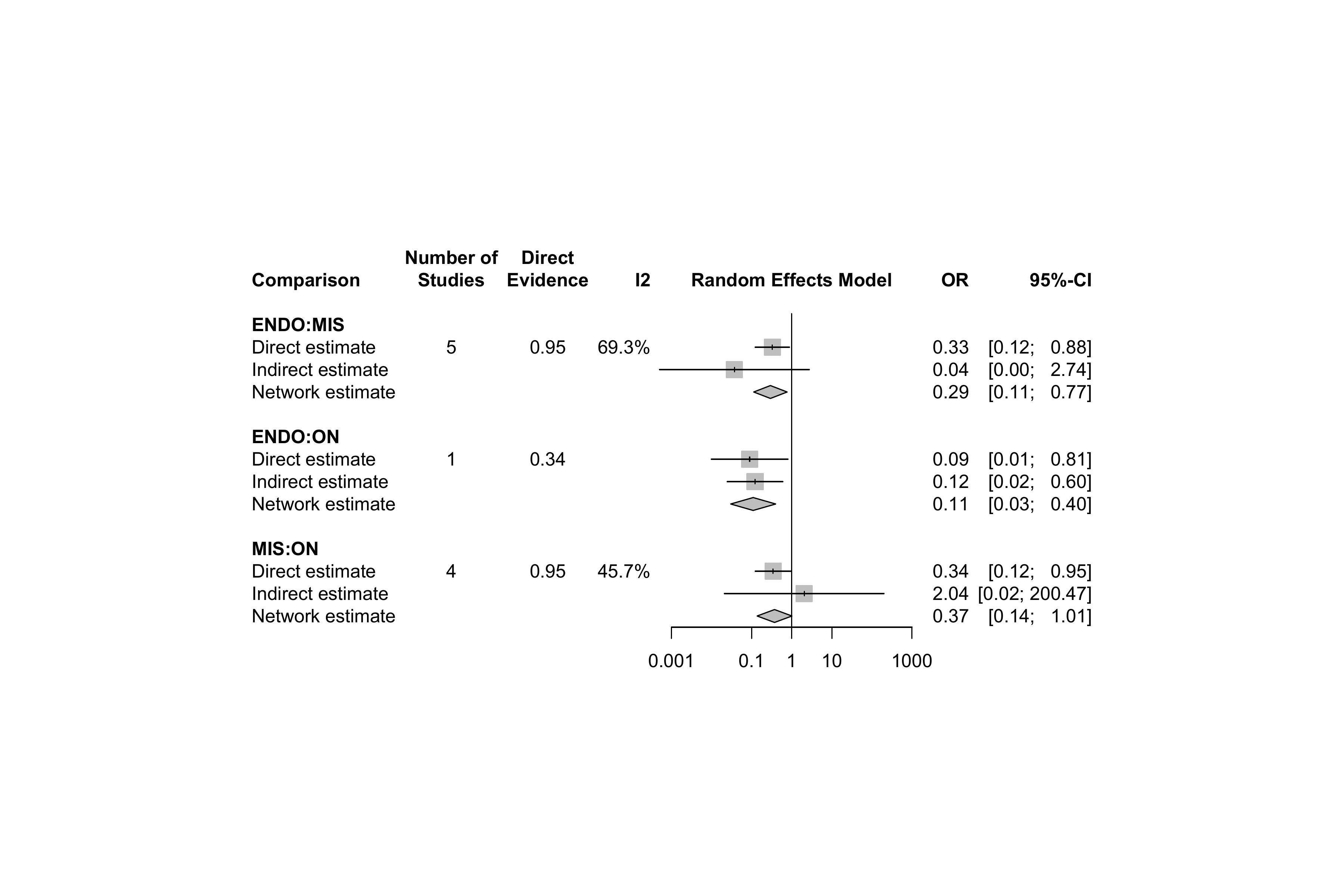


*Figure S2B. Node-splitting analysis for complications. Direct and indirect estimates compared. No significant inconsistency detected.*

## Figure S2C. Node-Split: New-Onset MOF


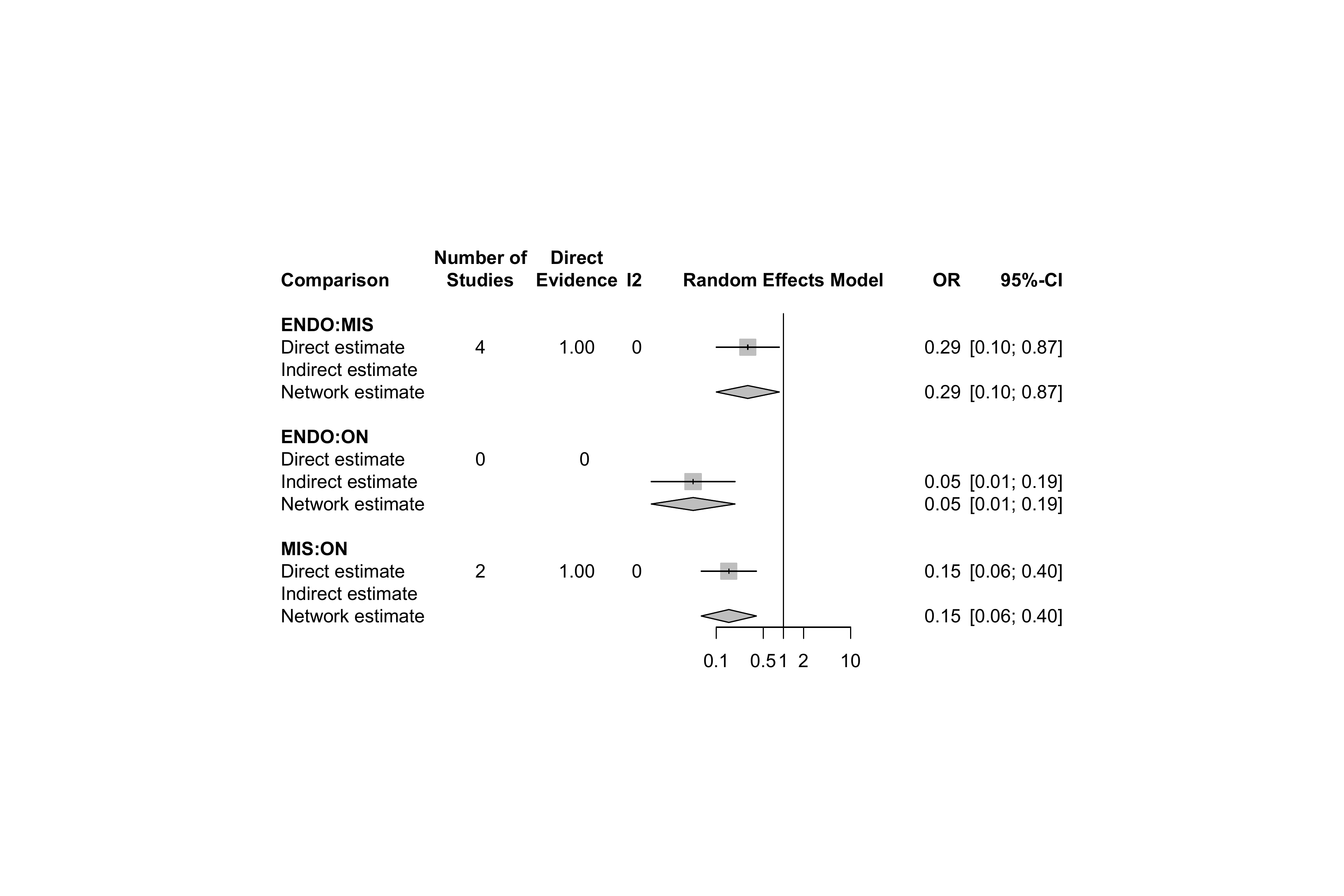


*Figure S2C. Node-splitting analysis for new-onset mof. Direct and indirect estimates compared. No significant inconsistency detected.*

## Figure S2D. Node-Split: Bleeding


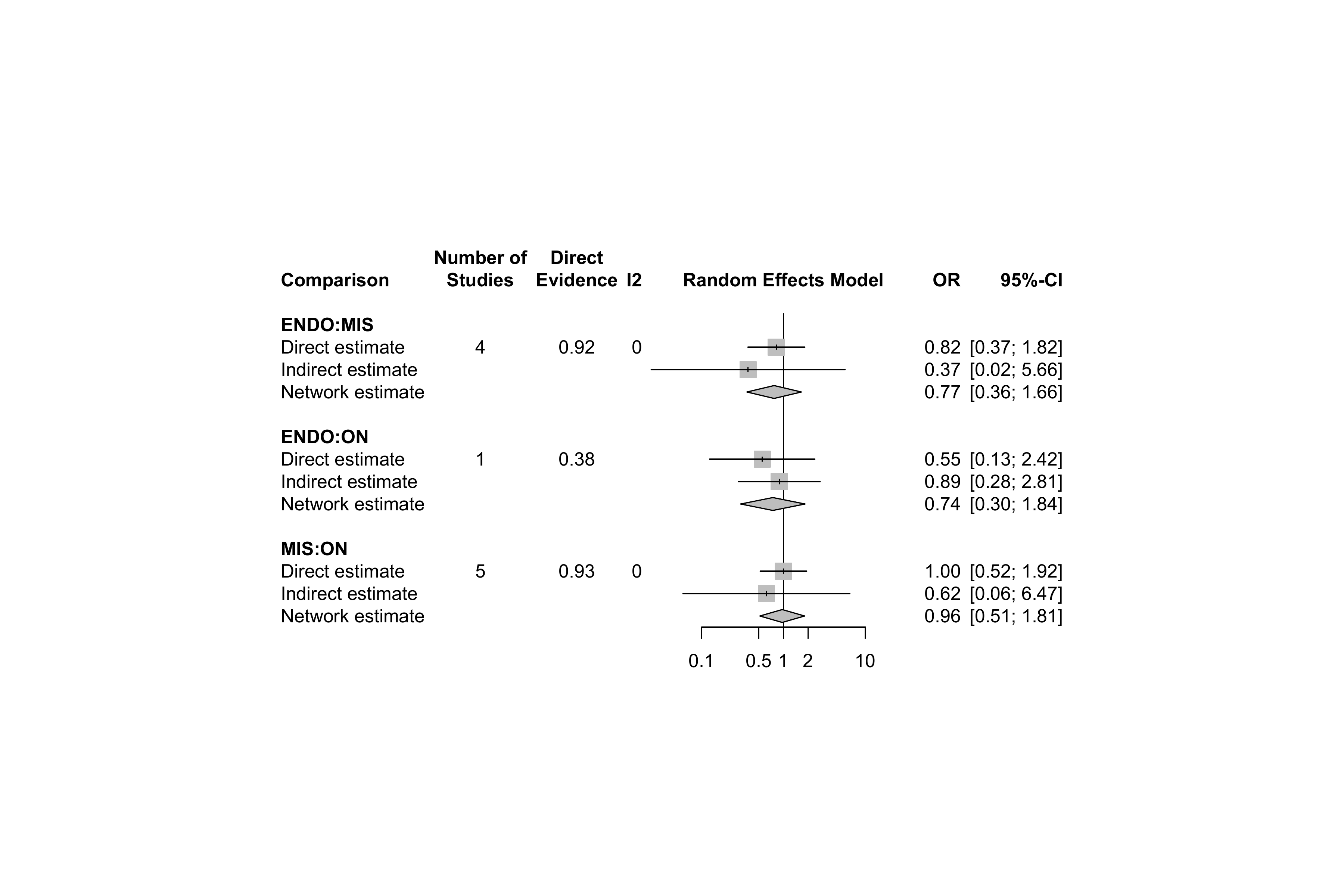


*Figure S2D. Node-splitting analysis for bleeding. Direct and indirect estimates compared. No significant inconsistency detected.*

## Figure S2E. Node-Split: New-Onset Diabetes


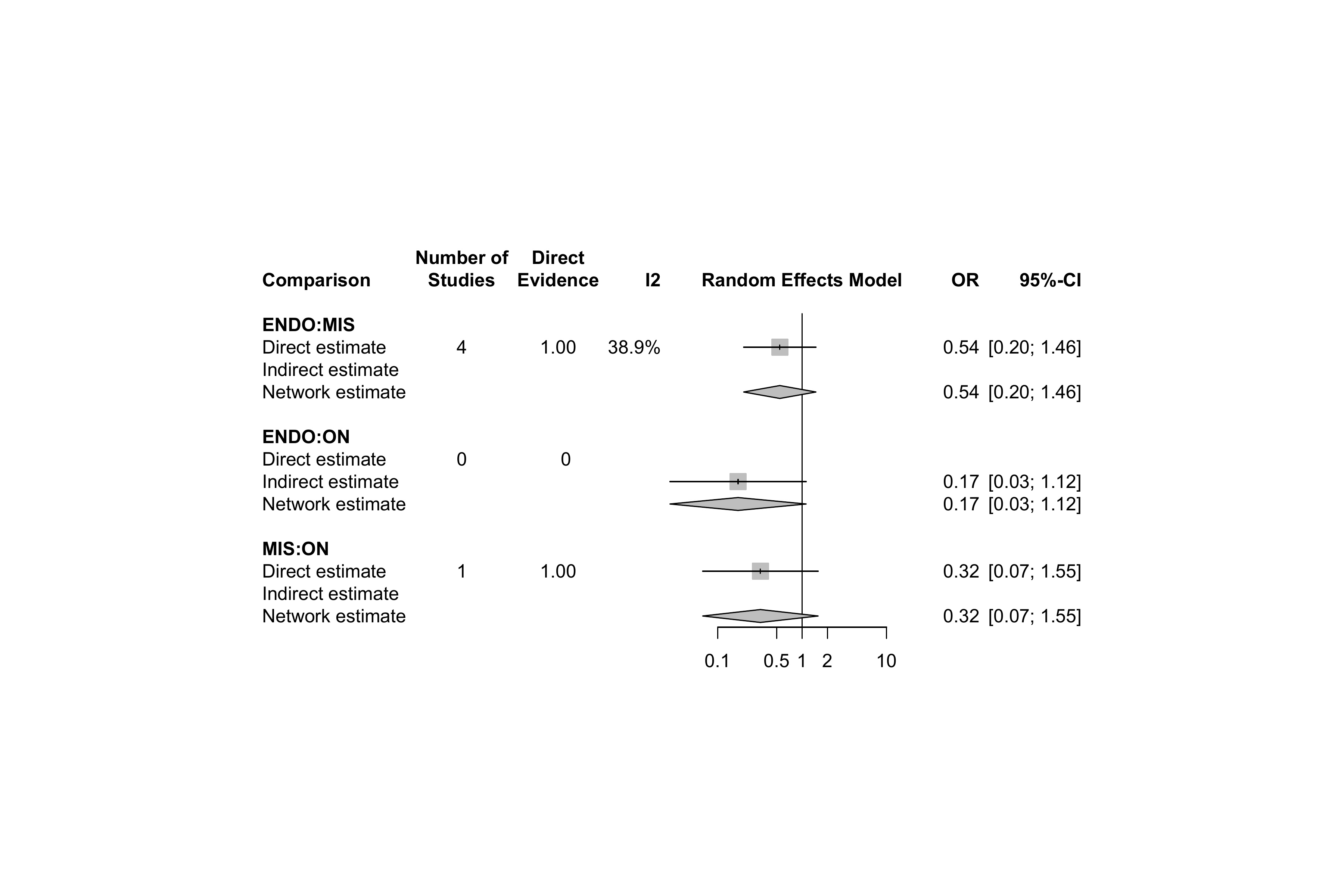


*Figure S2E. Node-splitting analysis for new-onset diabetes. Direct and indirect estimates compared. No significant inconsistency detected.*

## Figure S2F. Node-Split: Exocrine Insufficiency


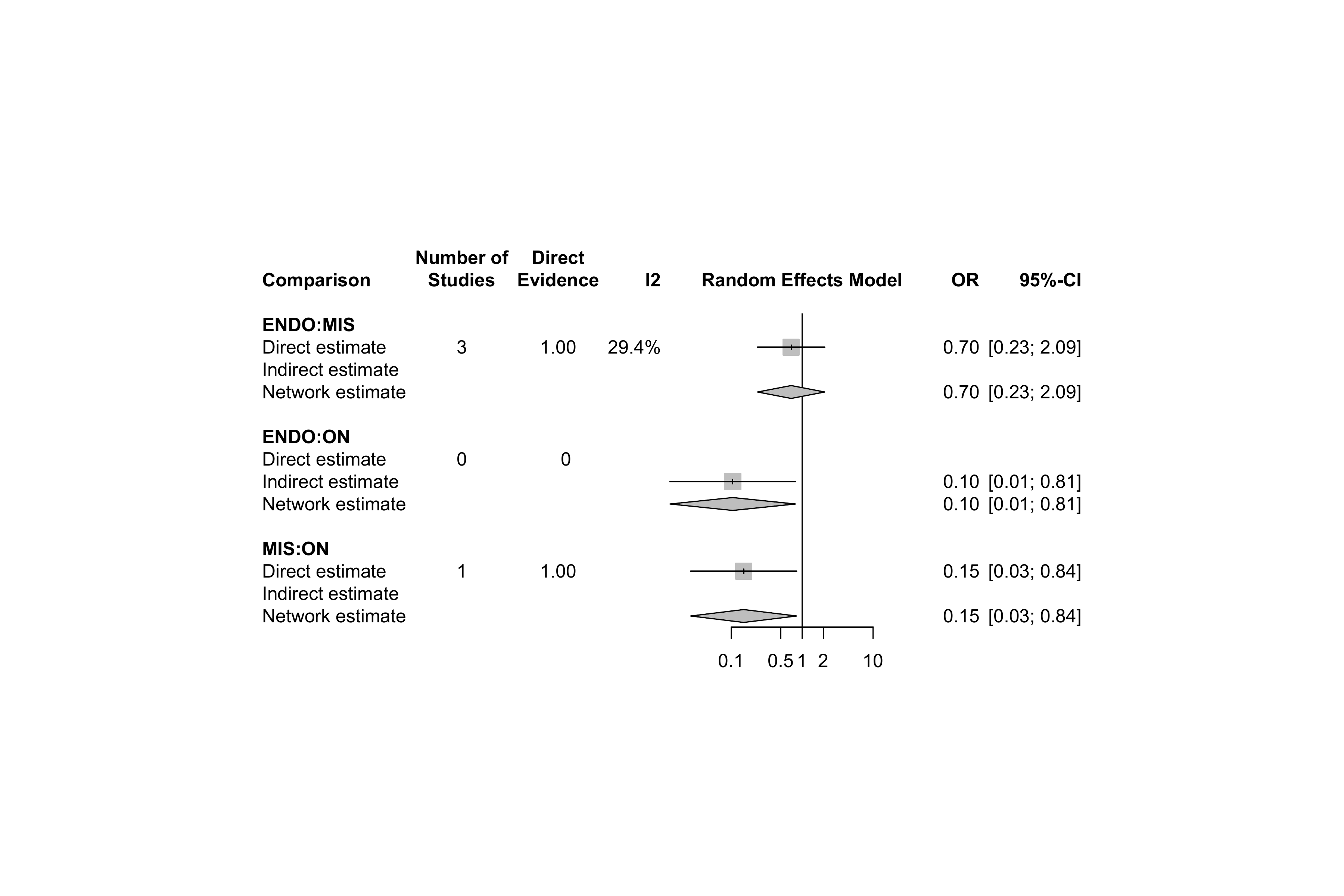


*Figure S2F. Node-splitting analysis for exocrine insufficiency. Direct and indirect estimates compared. No significant inconsistency detected.*

## Figure S2G. Node-Split: Reintervention


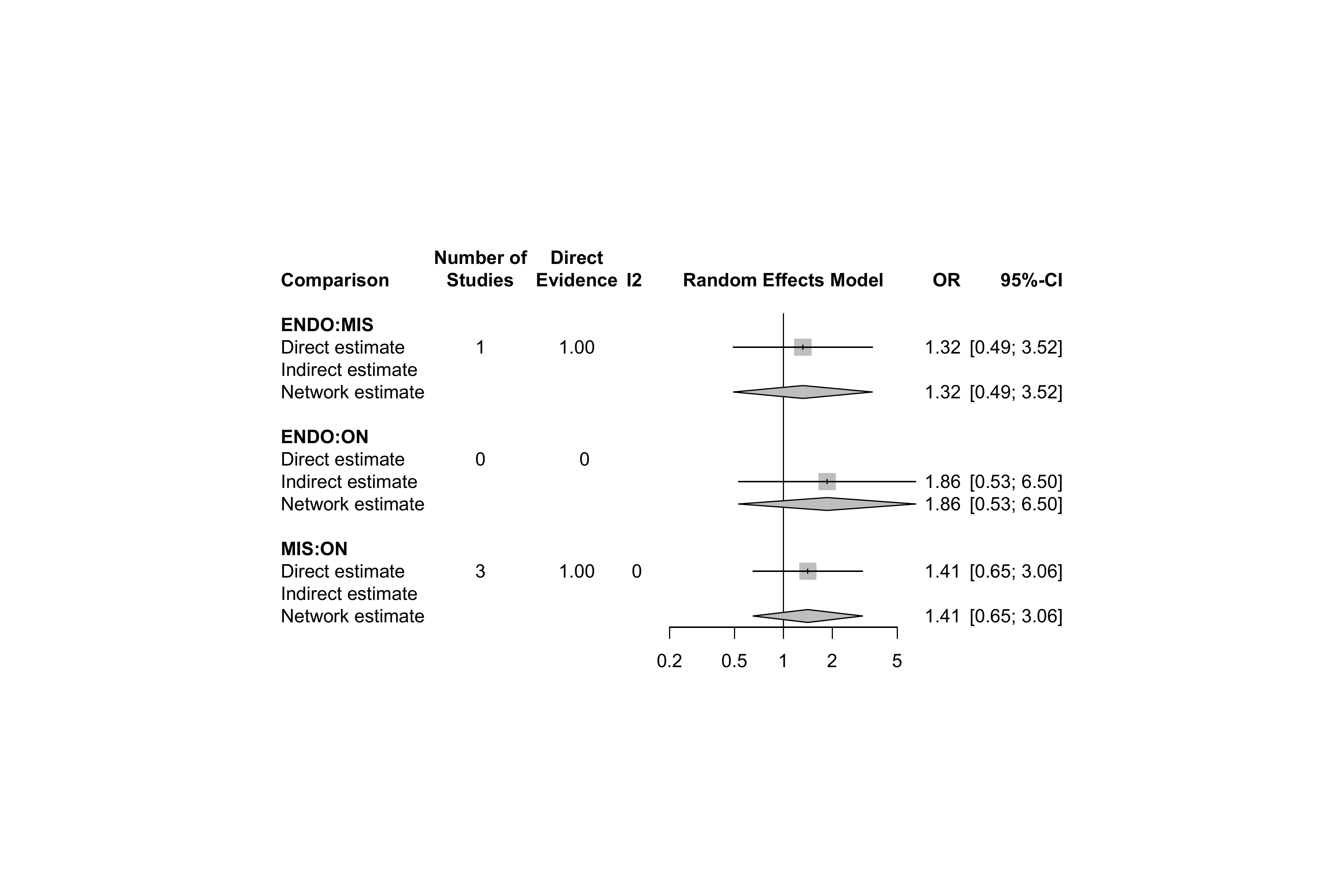


*Figure S2G. Node-splitting analysis for reintervention. Direct and indirect estimates compared. No significant inconsistency detected.*

## Figure S2H. Node-Split: Incisional Hernia


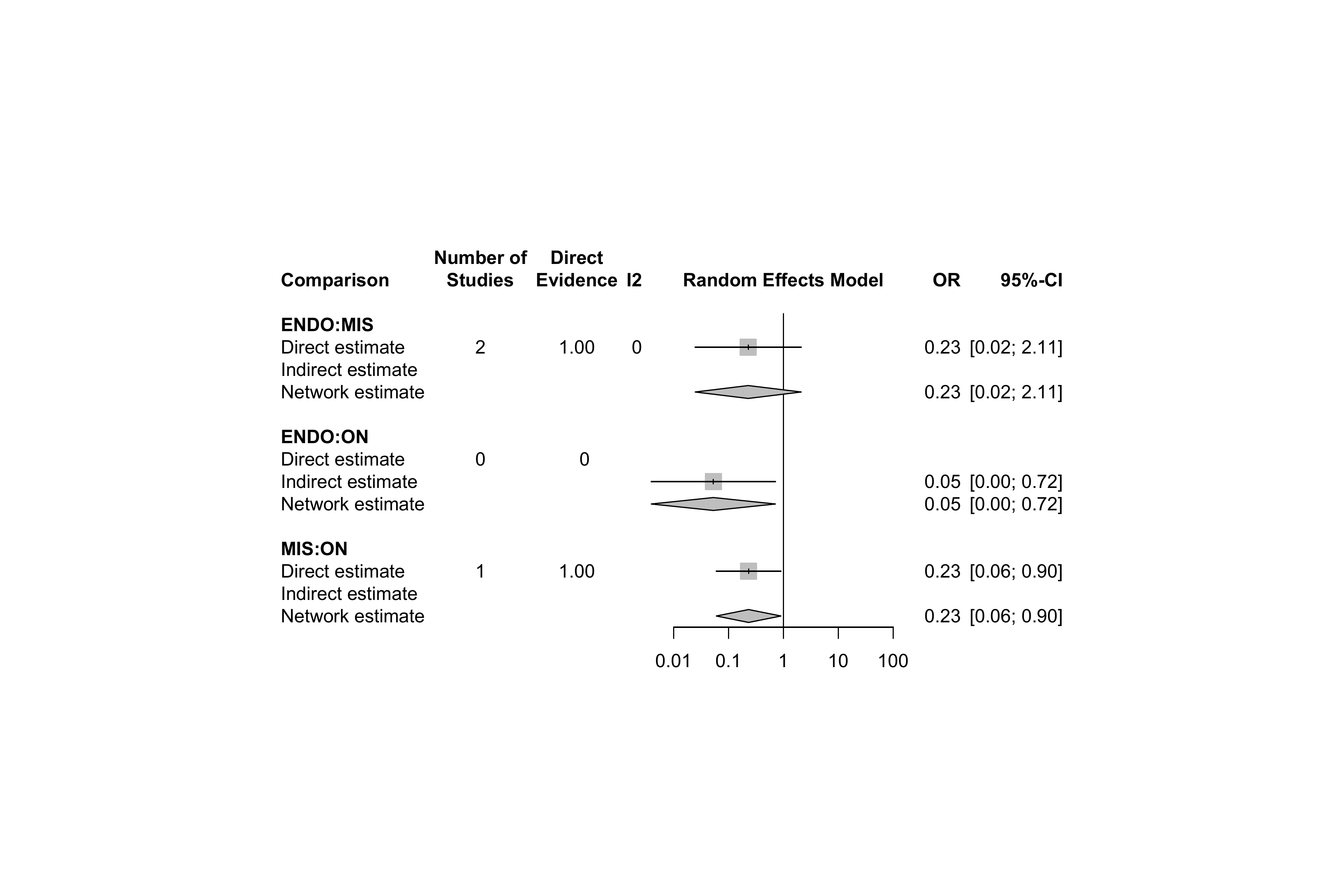


*Figure S2H. Node-splitting analysis for incisional hernia. Direct and indirect estimates compared. No significant inconsistency detected.*

## Figure S2I. Node-Split: New ICU Admission


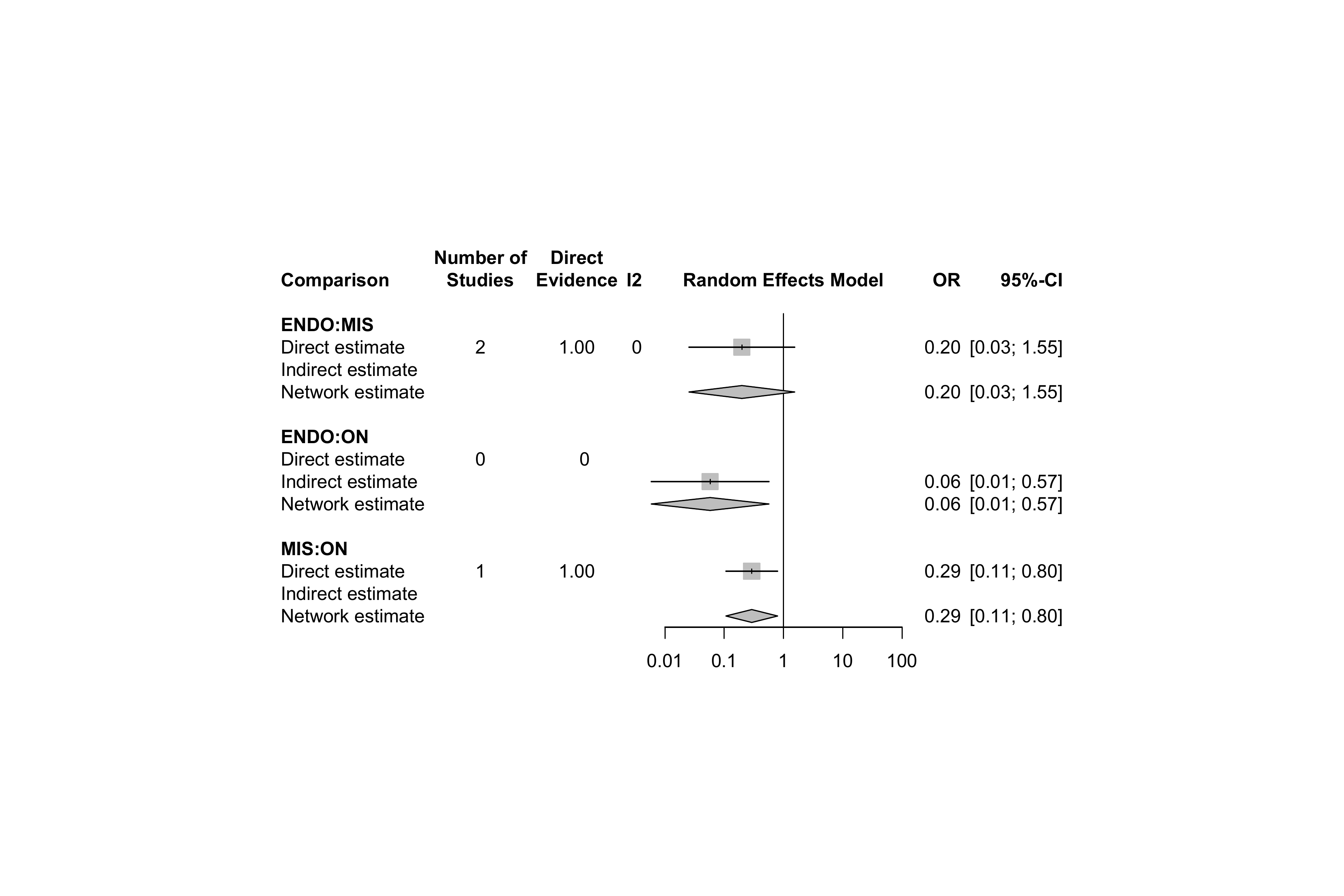


*Figure S2I. Node-splitting analysis for new icu admission. Direct and indirect estimates compared. No significant inconsistency detected.*

## Figure S3. Comparison-Adjusted Funnel Plots

Visual assessment of publication bias.

## Figure S3A. Funnel: Mortality


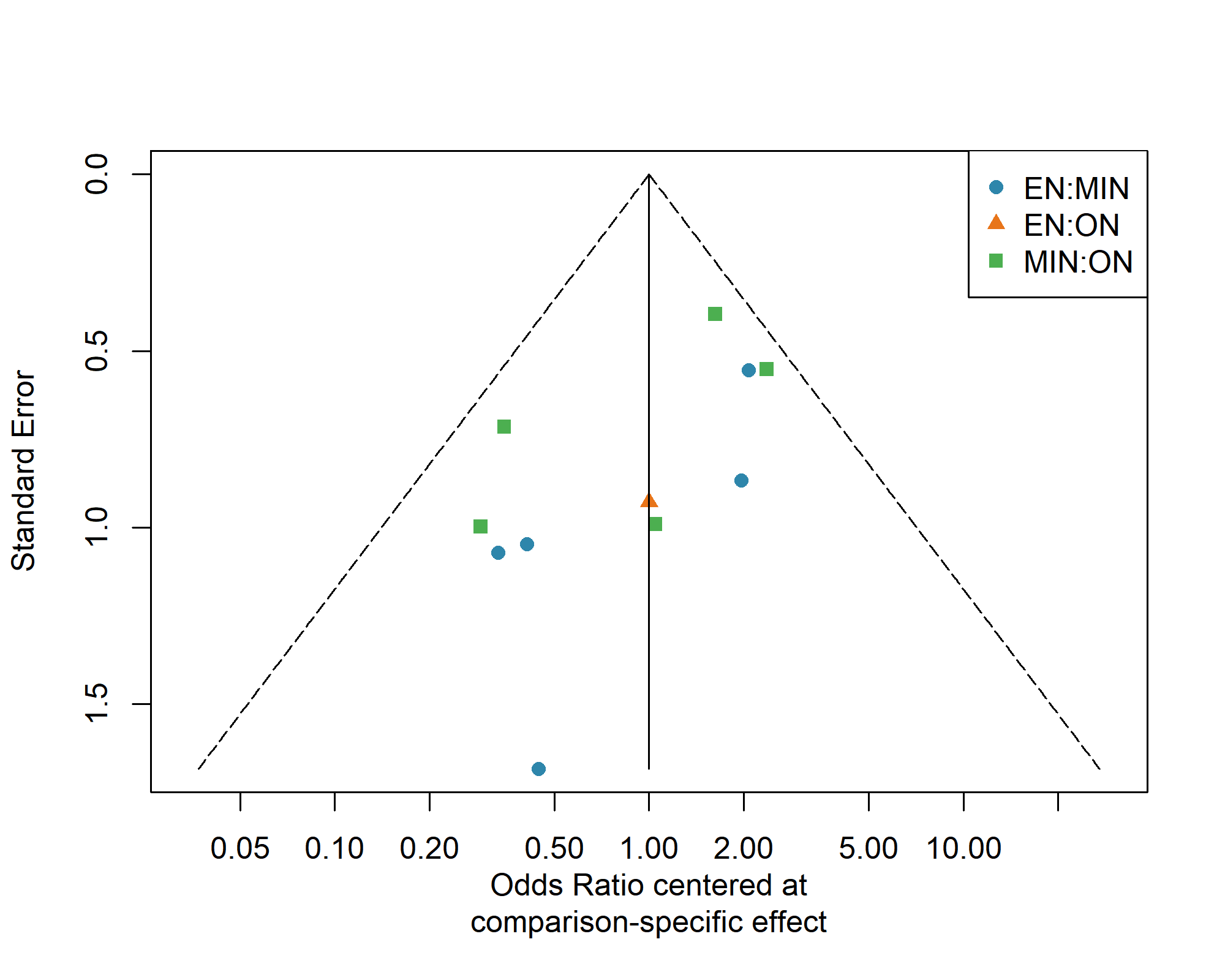


*Figure S3A. Comparison-adjusted funnel plot for mortality. No major asymmetry detected.*

## Figure S3B. Funnel: Complications


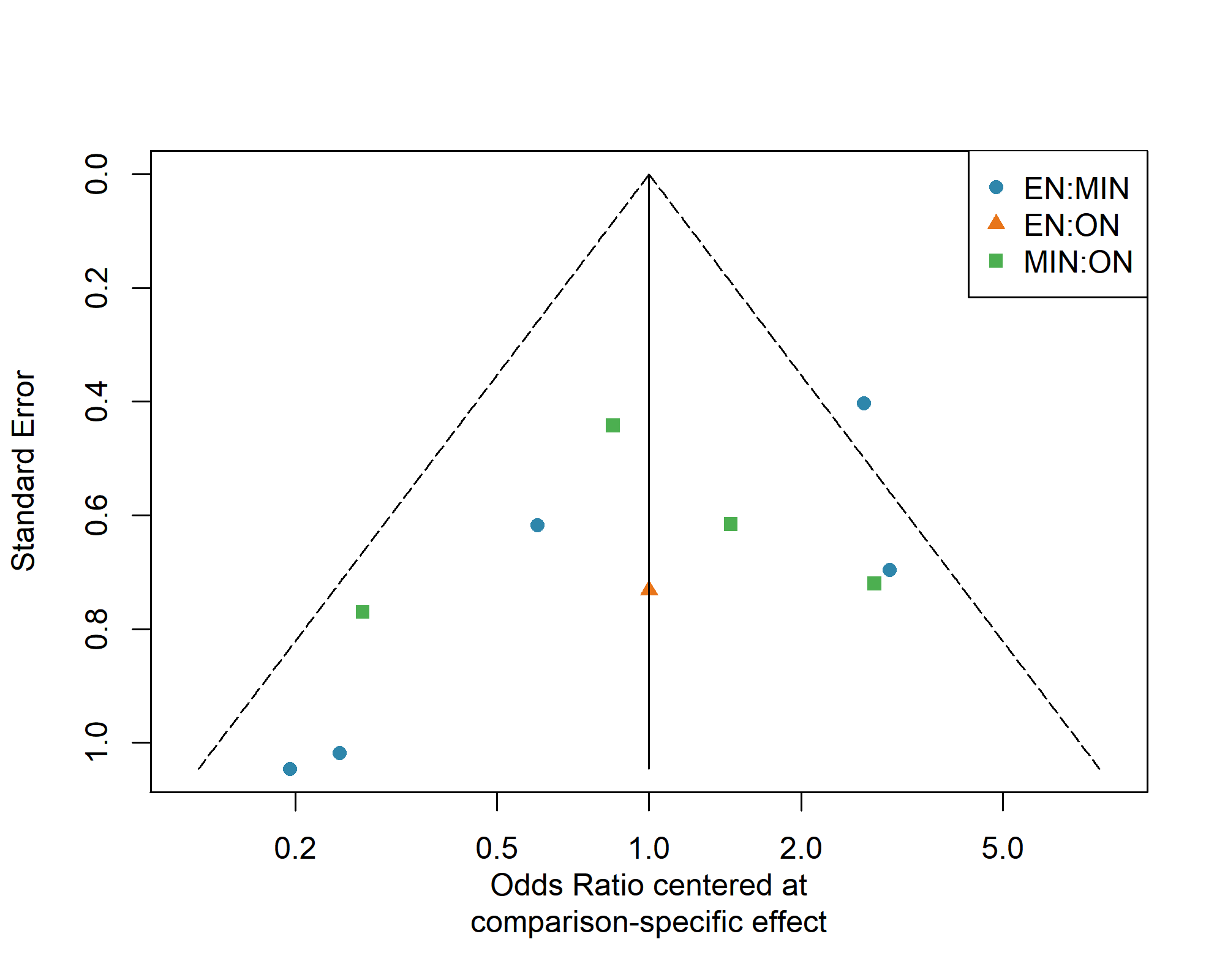


*Figure S3B. Comparison-adjusted funnel plot for complications. No major asymmetry detected.*

## Figure S3C. Funnel: New-Onset MOF


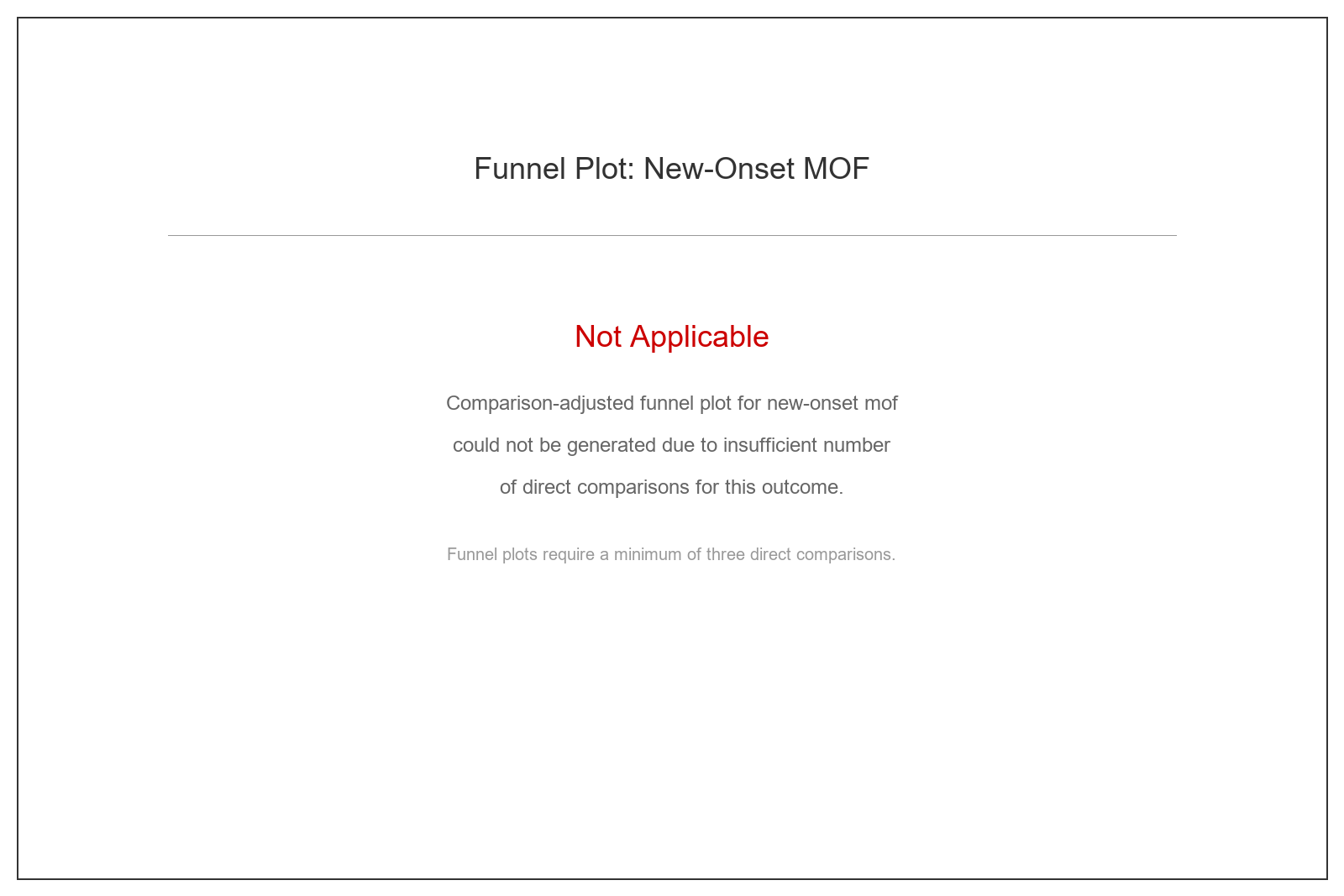


*Figure S3C. Comparison-adjusted funnel plot for new-onset mof. No major asymmetry detected.*

## Figure S3D. Funnel: Bleeding


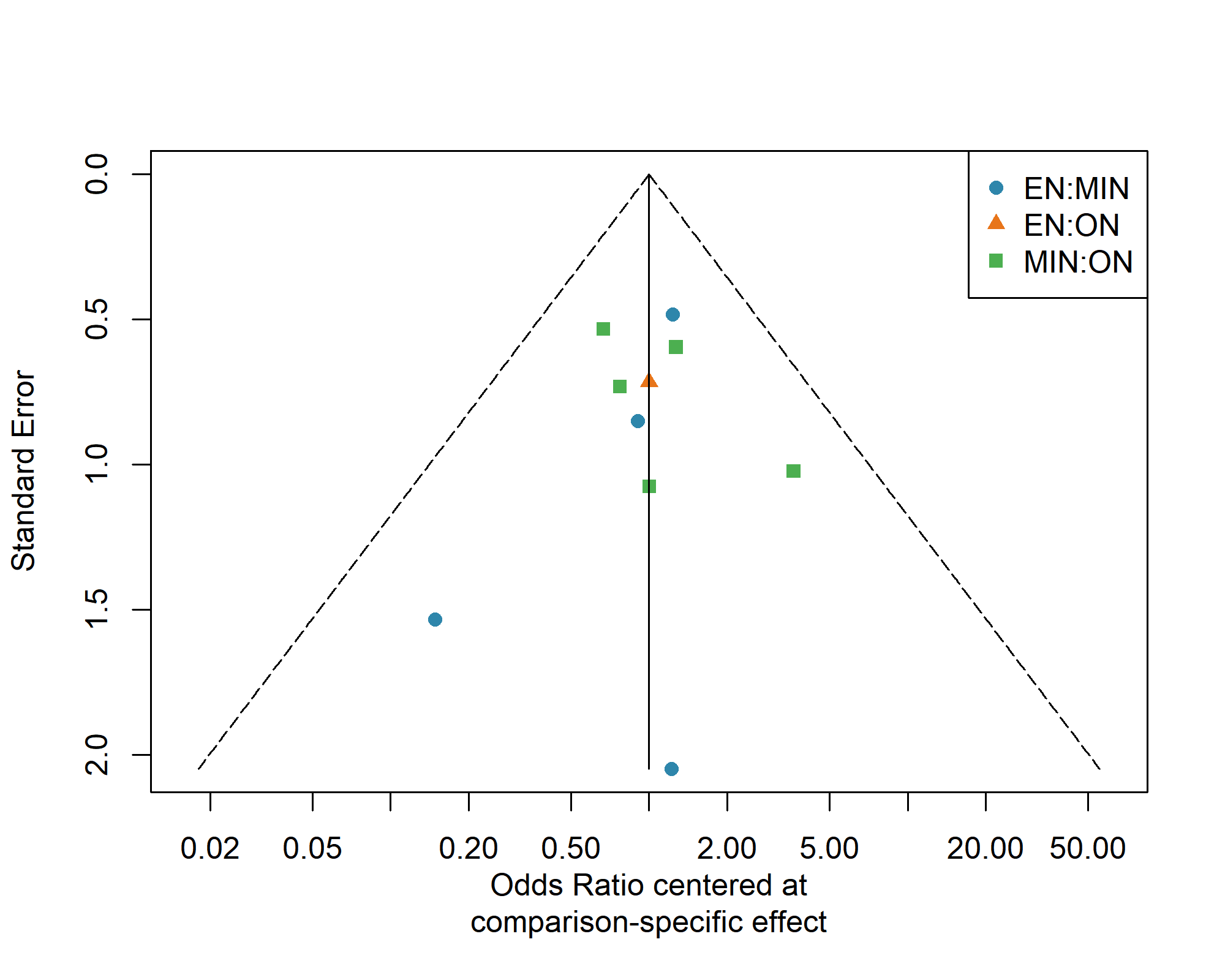


*Figure S3D. Comparison-adjusted funnel plot for bleeding. No major asymmetry detected.*

## Figure S3E. Funnel: New-Onset Diabetes


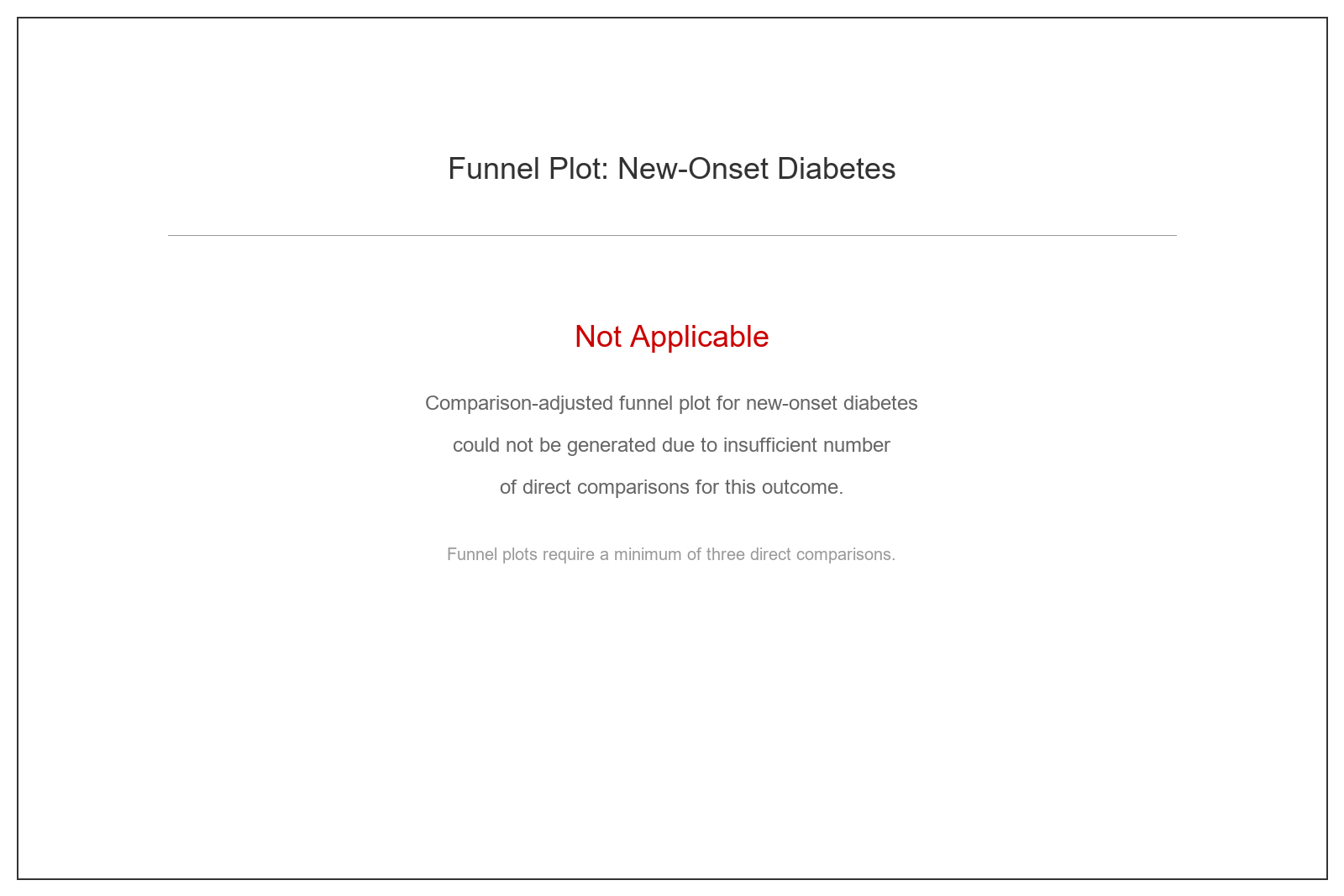


*Figure S3E. Comparison-adjusted funnel plot for new-onset diabetes. No major asymmetry detected.*

## Figure S3F. Funnel: Exocrine Insufficiency


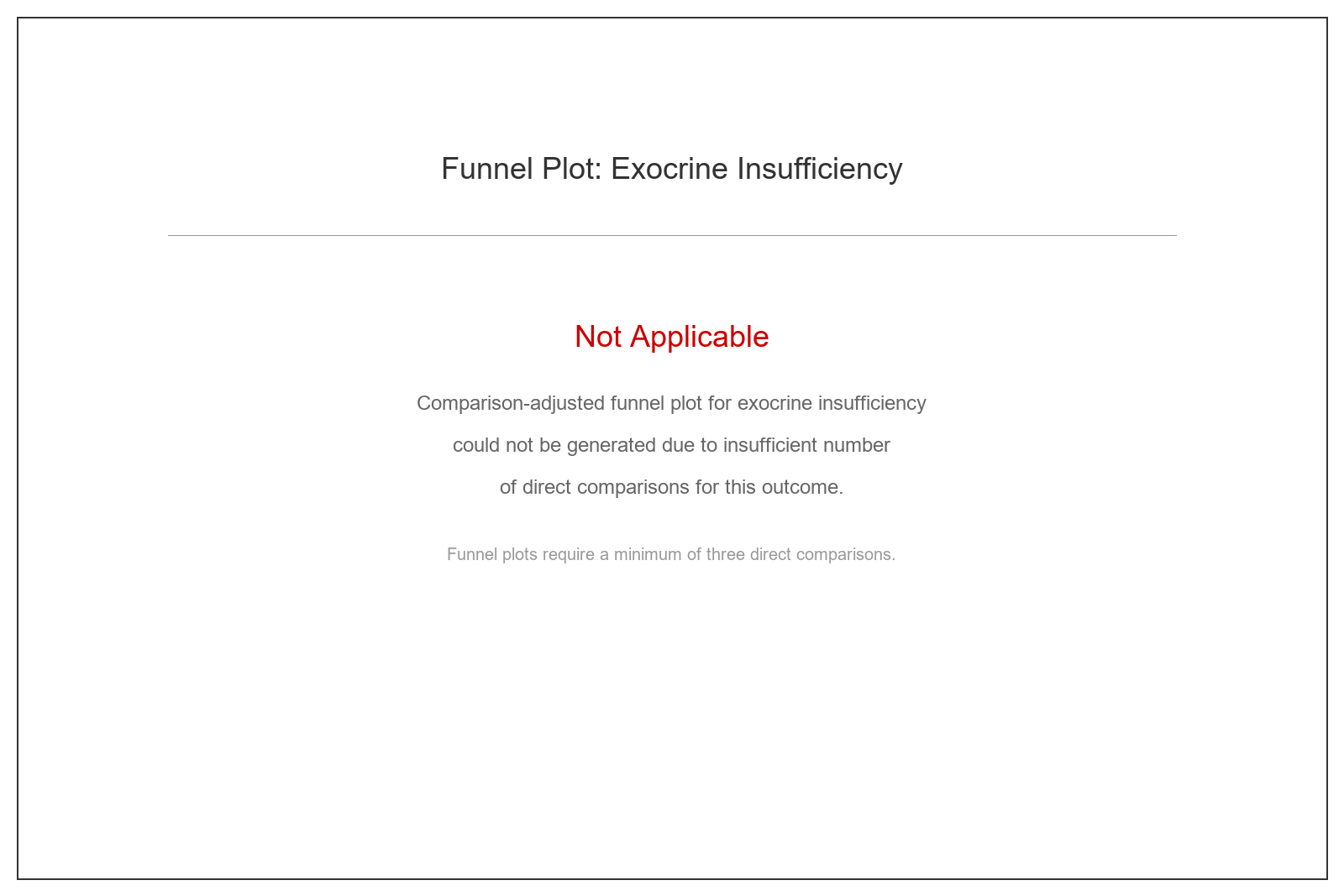


*Figure S3F. Comparison-adjusted funnel plot for exocrine insufficiency. No major asymmetry detected.*

## Figure S3G. Funnel: Reintervention


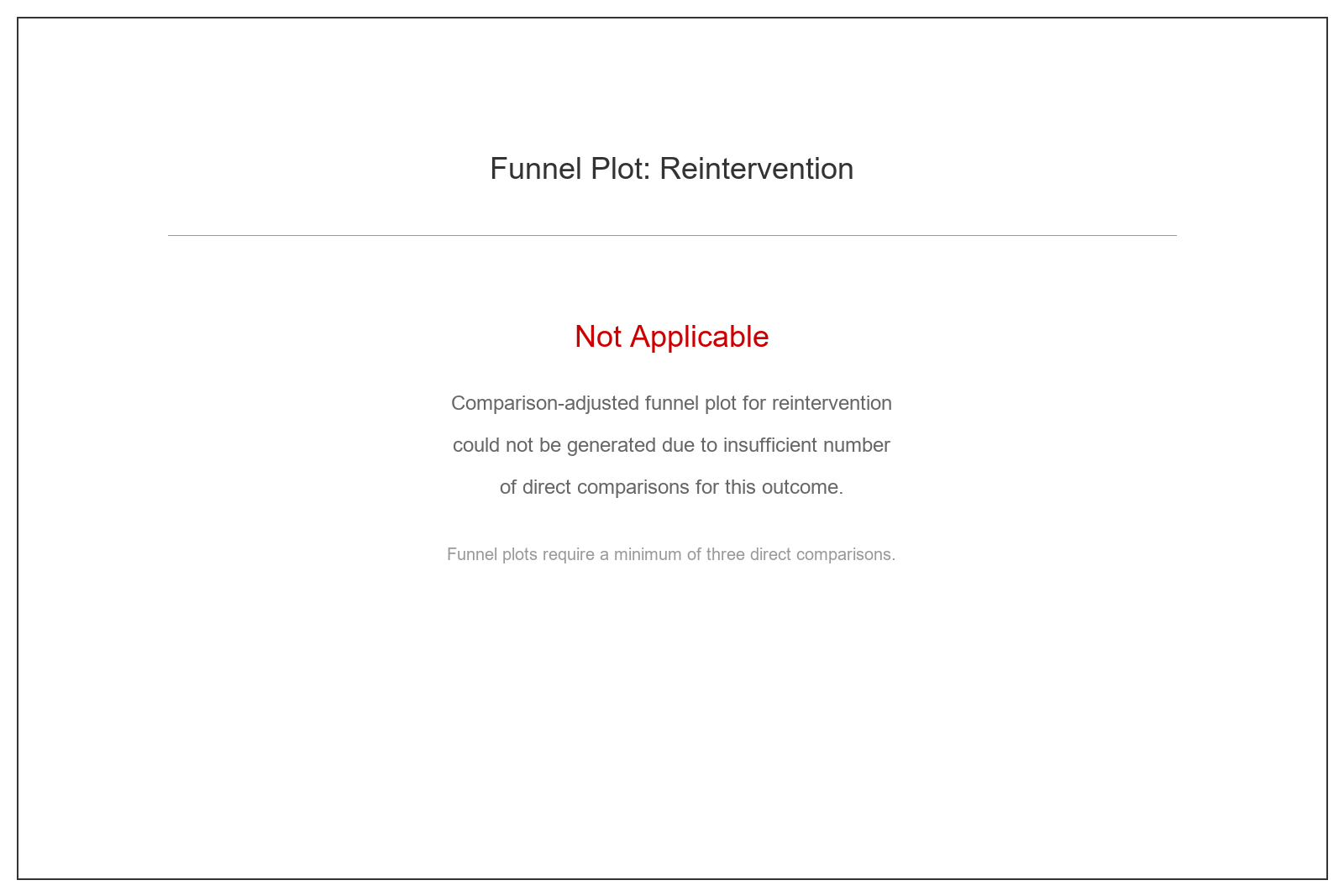


*Figure S3G. Comparison-adjusted funnel plot for reintervention. No major asymmetry detected.*

## Figure S3H. Funnel: Incisional Hernia


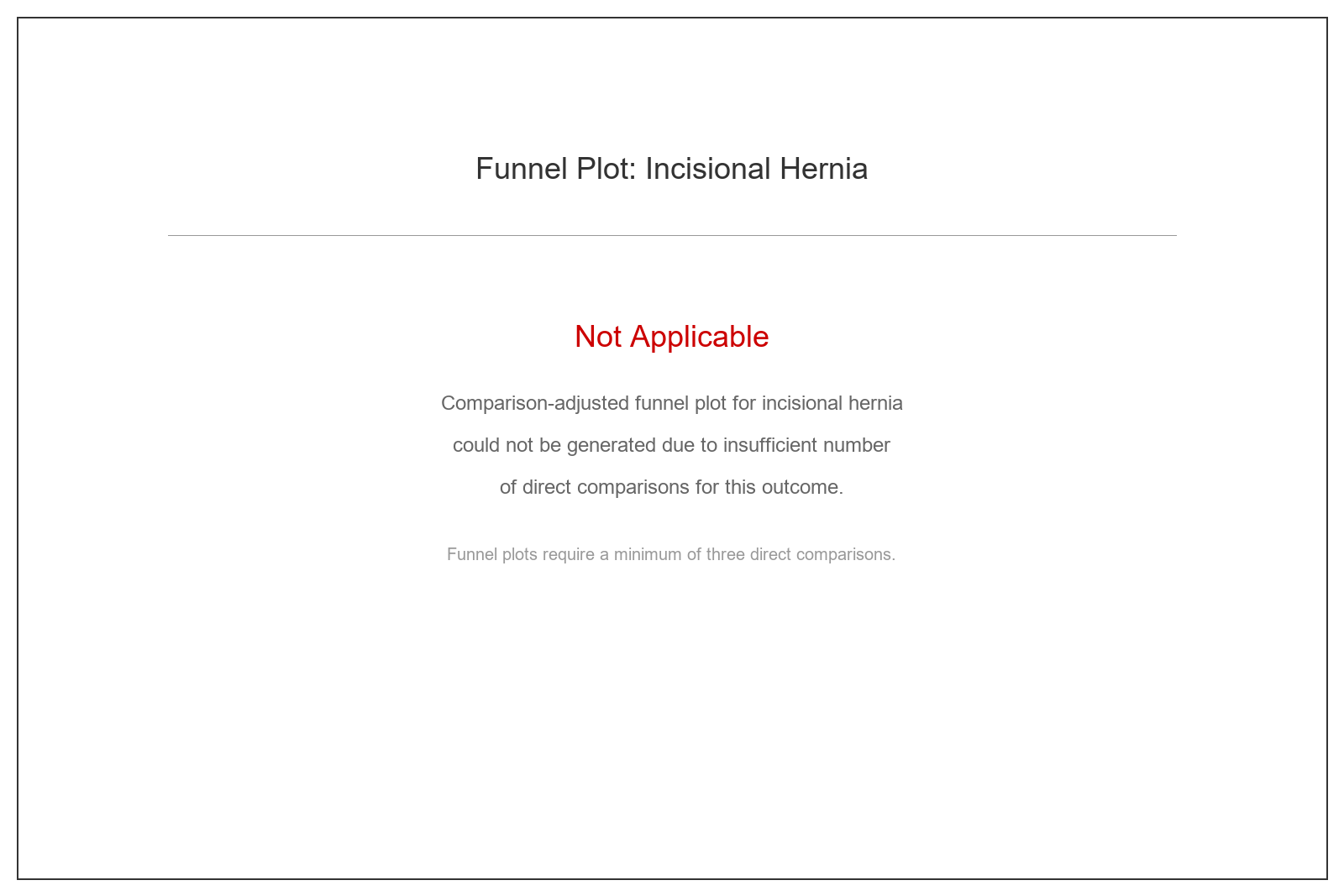


*Figure S3H. Comparison-adjusted funnel plot for incisional hernia. No major asymmetry detected.*

## Figure S3I. Funnel: New ICU Admission


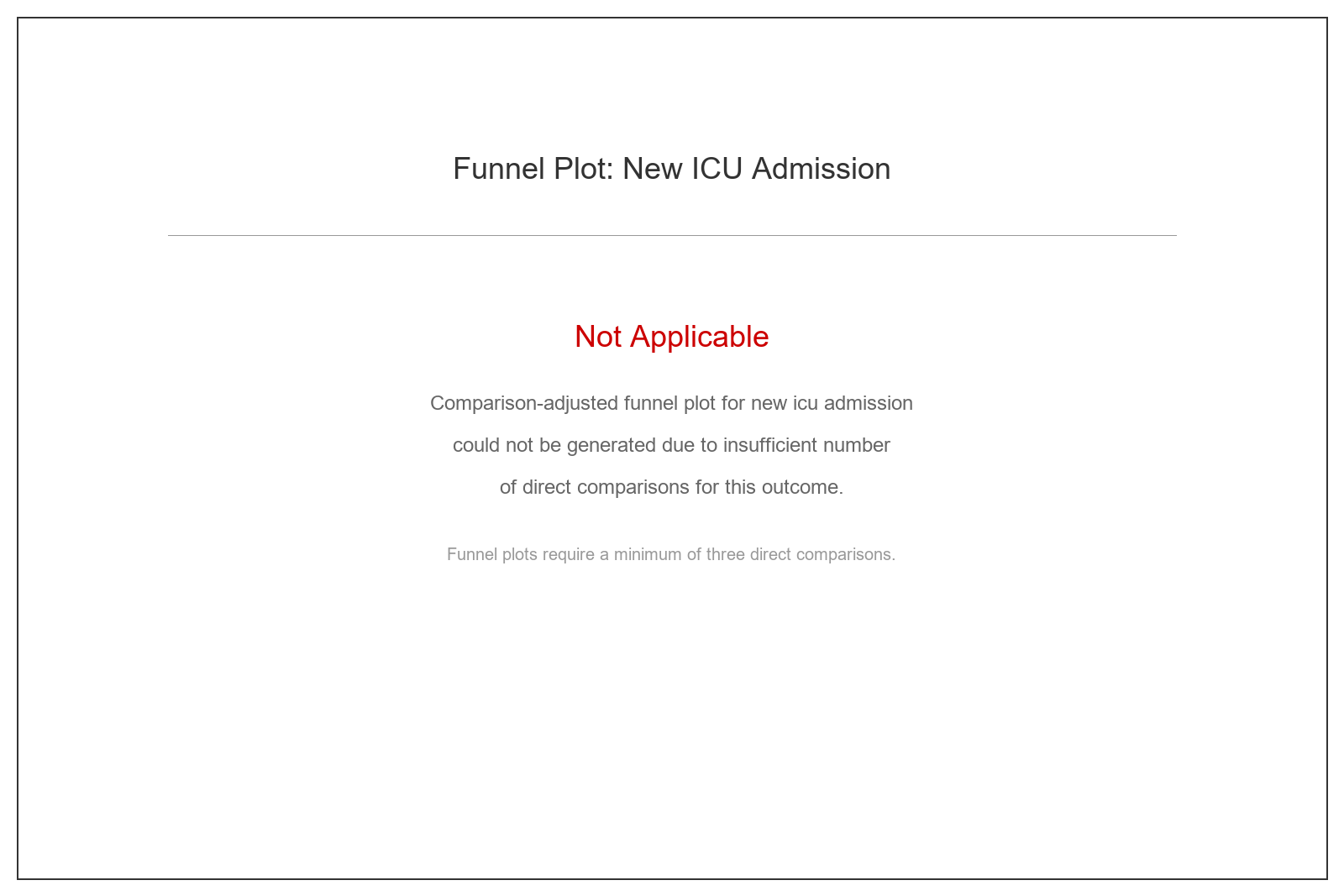


*Figure S3I. Comparison-adjusted funnel plot for new icu admission. No major asymmetry detected.*

## Figure S4. P-Score Heatmap


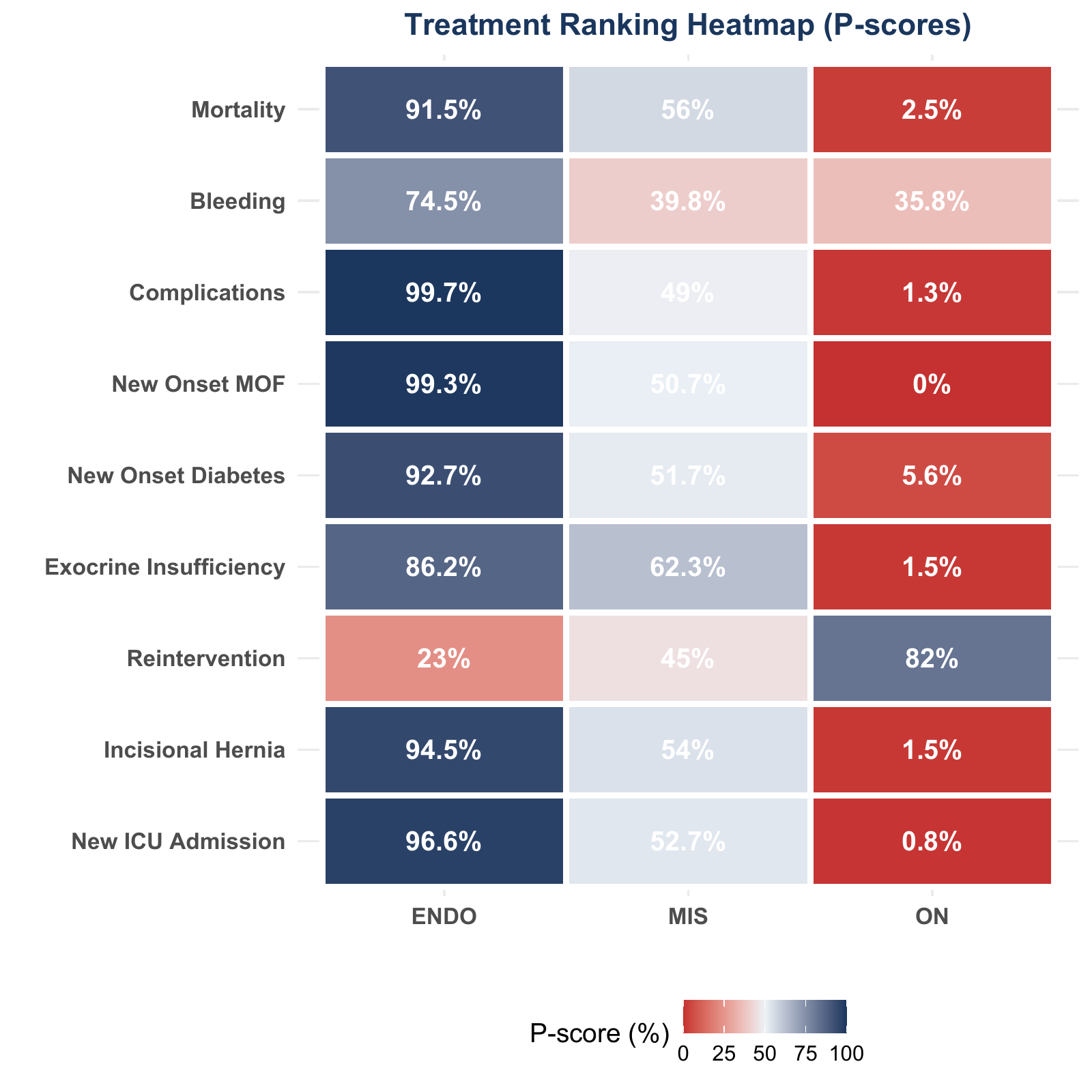


*Figure S4. P-score heatmap for all nine NMA outcomes. Green = high P-score (best); Red = low P-score (worst). EN consistently ranked best in 8/9 outcomes.*

## Figure S5. P-Score Bar Chart


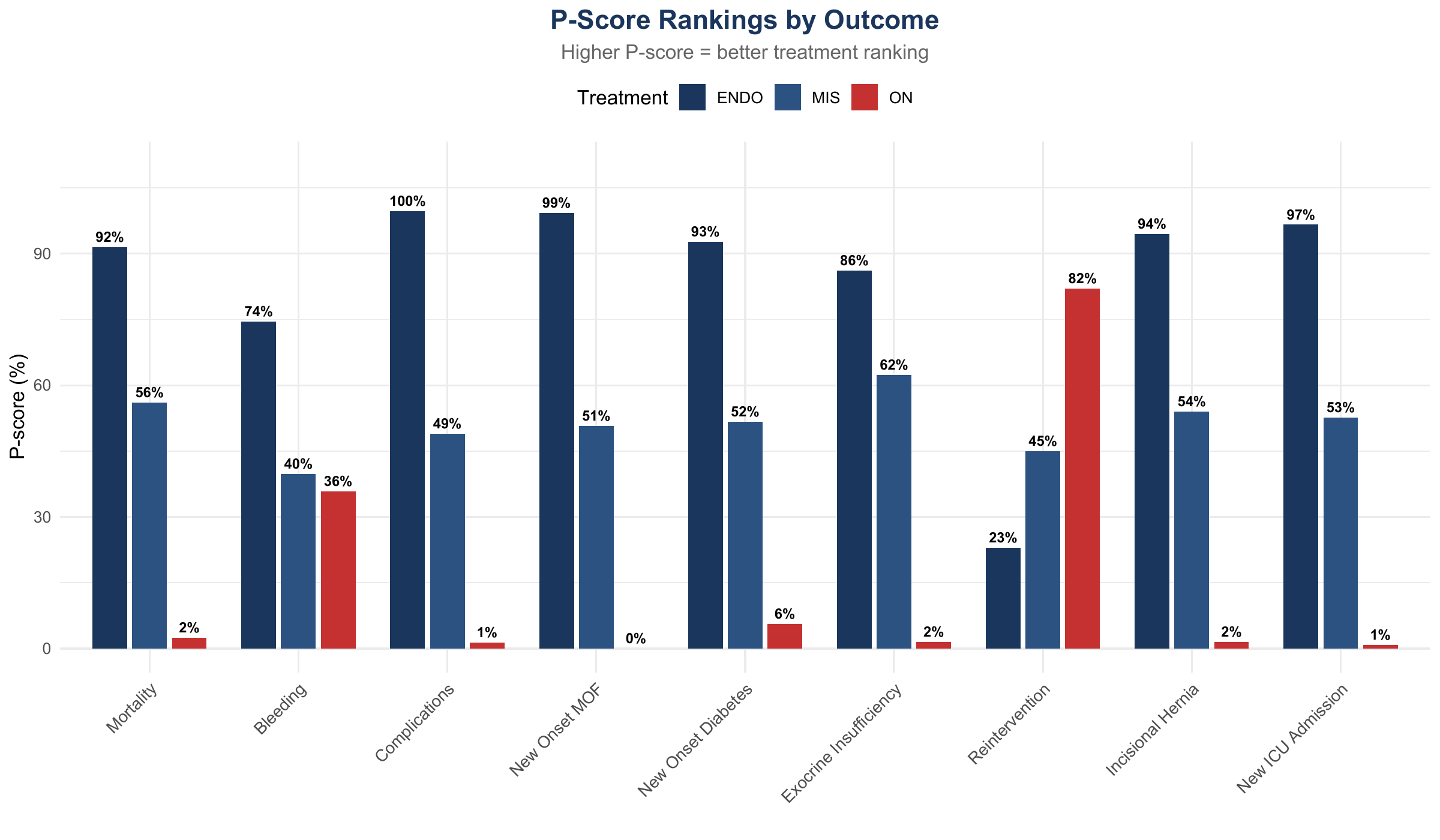


*Figure S5. P-score bar chart by treatment and outcome. EN (blue), MIN (orange), ON (navy).*

## Figure S6. Rankograms


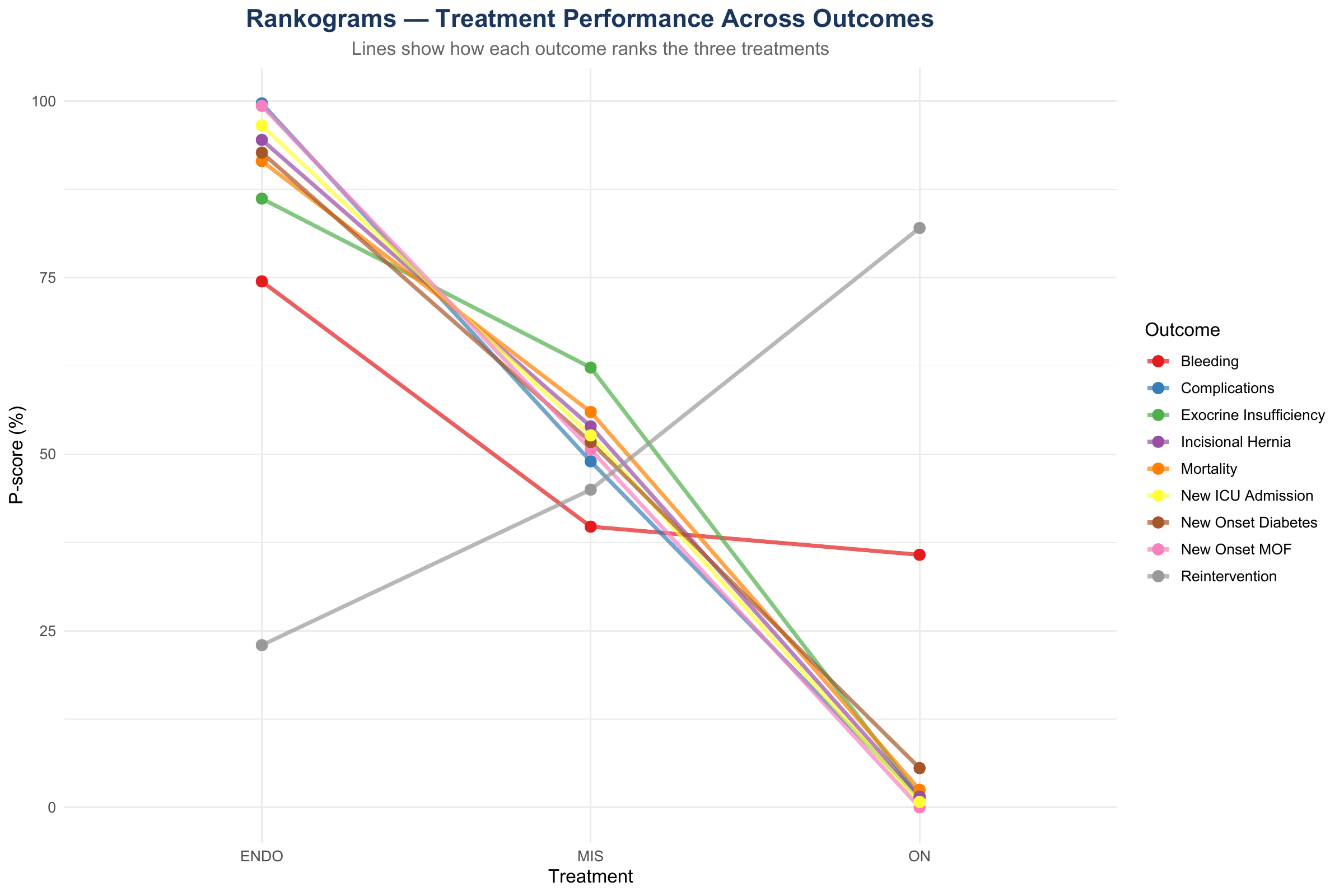


*Figure S6. Rankograms showing treatment ranking probabilities. EN (blue), MIN (orange), ON (navy).*
