# Supplementary material for: Comparative Outcomes of Endoscopic, Minimally Invasive Surgical, and Open Necrosectomy in Necrotizing Pancreatitis: Evidence From a Network Meta‐Analysis
Source: World J Surg. 2026 Jun 2;50(7):1875–90. doi: 10.1002/wjs.70406 (PMC13356536; doi:10.1002/wjs.70406)
Supplement: Supplementary file 3 — Supporting Information S2 [file WJS-50-1875-s001.docx]

**SUPPLEMENTARY TABLES**

## Supplementary Table S1. Sensitivity Analysis: New ICU Admission

Full NMA: EN vs ON OR = 0.06 [0.01-0.57]; MIN vs ON OR = 0.29 [0.11-0.80].

| **Study** | **Node 1** | **N** | **Events** | **Node 2** | **N** | **Events** |
| --- | --- | --- | --- | --- | --- | --- |
| vanSantvoort2010_PANTER | ON | 45 | 18 | MIN | 43 | 7 |
| Bakker2012_PENGUIN | MIN | 10 | 5 | EN | 10 | 1 |
| Bang2019_MISER | MIN | 32 | 0 | EN | 34 | 0 |

*Leave-one-out: excluding each study preserves EN and MIN superiority over ON.*

## Supplementary Table S2. GI Fistula — Study-Level Data

| **Study** | **Node 1** | **Events/N** | **Node 2** | **Events/N** |
| --- | --- | --- | --- | --- |
| vanSantvoort2010_PANTER | ON | 10/45 | MIN | 6/43 |
| Bakker2012_PENGUIN | MIN | 2/10 | EN | 0/10 |
| vanBrunschot2017_TENSION | MIN | 8/47 | EN | 4/51 |
| Bang2019_MISER | MIN | 9/32 | EN | 0/34 |
| Avudiappan2023 | ON | 10/78 | MIN | 2/44 |
| Bausch2012 | ON | 14/30 | MIN | 0/14 |
| Bausch2012 | ON | 14/30 | EN | 5/18 |
| Bausch2012 | MIN | 0/14 | EN | 5/18 |
| Tu2013 | ON | 2/32 | MIN | 0/18 |
| vanSantvoort2007 | ON | 2/15 | MIN | 1/15 |

*EN = endoscopic, MIN = minimally invasive, ON = open necrosectomy.*

## Supplementary Table S3. CINeMA Confidence Assessment

| **Comparison** | **Outcome** | **Studies** | **Within_Study_Bias** | **Indirectness** | **Imprecision** | **Heterogeneity** | **Incoherence** | **Publication_Bias** | **Confidence** |
| --- | --- | --- | --- | --- | --- | --- | --- | --- | --- |
| All vs ON | Mortality | 9 | Some concerns | Low | Some concerns | Some concerns | Some concerns | Undetected | Low |
| All vs ON | Complications | 8 | Low | Low | Some concerns | Major | Some concerns | Undetected | Low |
| All vs ON | New Onset MOF | 6 | Low | Low | Some concerns | Low | Low | Undetected | Moderate |
| All vs ON | Bleeding | 8 | Low | Low | Some concerns | Low | Low | Undetected | Moderate |
| All vs ON | New Onset Diabetes | 5 | Low | Low | Major | Some concerns | Low | Undetected | Low |
| All vs ON | Exocrine Insufficiency | 4 | Low | Low | Major | Some concerns | Low | Undetected | Low |
| All vs ON | Reintervention | 4 | Some concerns | Low | Some concerns | Low | Low | Undetected | Low |
| All vs ON | Incisional Hernia | 3 | Low | Low | Major | Low | Low | Undetected | Low |
| All vs ON | New ICU Admission | 3 | Low | Low | Major | Low | Low | Undetected | Low |

*CINeMA framework (Nikolakopoulou et al., 2020).*
